# Structural basis of Zika virus NS1 multimerization and human antibody recognition

**DOI:** 10.1038/s44298-024-00024-6

**Published:** 2024-04-25

**Authors:** Bing Liang Alvin Chew, An Qi Ngoh, Wint Wint Phoo, Mei Jie Grace Weng, Ho Jun Sheng, Kitti Wing Ki Chan, Eddie Yong Jun Tan, Terri Gelbart, Chenrui Xu, Gene S. Tan, Subhash G. Vasudevan, Dahai Luo

**Affiliations:** 1 Lee Kong Chian School of Medicine, Nanyang Technological University, Singapore, Singapore; 2 NTU Institute of Structural Biology, Nanyang Technological University, Singapore, Singapore; 3 Program in Emerging Infectious Diseases, Duke-NUS Medical School, Singapore, Singapore; 4 School of Biological Sciences, Nanyang Technological University, Singapore, Singapore; 5 Infectious Diseases, The J. Craig Venter Institute, La Jolla, CA USA; 6 Division of Infectious Diseases and Global Public Health, Department of Medicine, University of California San Diego, La Jolla, CA USA; 7 Department of Microbiology and Immunology, National University of Singapore, Singapore, Singapore; 8 Institute for Glycomics (G26), Griffith University Gold Coast Campus, Southport, QLD Australia; 9 National Centre for Infectious Diseases, Singapore, Singapore

**Keywords:** Dengue virus, Virus-host interactions, Microbiology

## Abstract

Zika virus (ZIKV) belongs to the *Flavivirus* genus of the *Flaviviridae* family along with the four serotypes of dengue virus (DENV1–4). The recent global outbreaks of contemporary ZIKV strains demonstrated that infection can lead to neurological sequelae in adults and severe abnormalities in newborns that were previously unreported with ancestral strains. As such, there remains an unmet need for efficacious vaccines and antiviral agents against ZIKV. The non-structural protein 1 (NS1) is secreted from the infected cell and is thought to be associated with disease severity besides its proven usefulness for differential diagnoses. However, its physiologically relevant structure and pathogenesis mechanisms remain unclear. Here, we present high-resolution cryoEM structures of ZIKV recombinant secreted NS1 (rsNS1) and its complexes with three human monoclonal antibodies (AA12, EB9, GB5), as well as evidence for ZIKV infection-derived secreted NS1 (isNS1) binding to High Density Lipoprotein (HDL). We show that ZIKV rsNS1 forms tetramers and filamentous repeats of tetramers. We also observed that antibody binding did not disrupt the ZIKV NS1 tetramers as they bound to the wing and connector subdomain of the β-ladder. Our study reveals new insights into NS1 multimerization, highlights the need to distinguish the polymorphic nature of rsNS1 and isNS1, and expands the mechanistic basis of the protection conferred by antibodies targeting NS1.

## Introduction

Zika virus (ZIKV) belongs to the *Flavivirus* genus, which includes other anthropod-borne viruses which can cause severe human diseases, such as the four serotypes of dengue virus (DENV1–4) and West Nile virus (WNV). ZIKV is transmitted by Aedes mosquito and has re-emerged in recent ZIKV epidemics, with previously unreported cases of congenital microcephaly and Guillain–Barré syndrome in adults. ZIKV remains a public health threat^1,2^ with no effective antivirals or vaccines available.

One therapeutic or vaccine target is the virus-encoded multifunctional non-structural protein 1 (NS1). NS1 can be found intracellularly, where it is essential for virus replication^3–5^, docked on lipid rafts of the cell surface membrane^6^, and abundantly secreted into the bloodstream of infected individuals during the febrile phase^7^. Interestingly, studies have shown that secreted NS1 (sNS1) has a pathophysiological influence on tissue tropism because of its different surface charge profiles^8,9^. Earlier efforts by our group and others have shown that NS1-targeted monoclonal antibodies (mAbs) are protective in vivo either through Fcγ-dependent^10,11^ and/or Fcγ-independent pathway^12–15^, without the known antibody-dependent enhancement effect of envelope protein-targeted mAbs. We also showed that an NS1-based ZIKV vaccine can inhibit pathogenicity in vivo^16^. However, autoimmunity concerns regarding NS1-targeted mAbs hinder vaccine development strategies as reviewed^17–19^, underscoring the need for complete structural details that underpin antibody-dependent protection.

Unraveling how antibodies recognize and bind to their epitopes is crucial for delineating the mechanisms by which antibodies protect against disease. While a large body of work has focused on studying neutralizing antibodies against the flavivirus envelope (E), there is still a gap in knowledge concerning the role and mechanisms by which NS1 antibodies provide protection. NS1 is comprised of three distinct domains, an N-terminal hydrophobic β-roll dimerization domain (residues 1–29) formed by the intertwining of two protomers, an α/β-wing domain (residues 38–151) that extends out from the central β-ladder domain (residues 181–352) giving the dimer a distinct crossed shape^8,20,21^. The connector segments between the three domains, residues 30–37 and 152–180, form a 3-stranded β-sheet. The NS1 has a membrane-associated face that features a flexible loop (residues 108–129) and the “greasy finger” (residues 159–163) of the wing domain, and the β-strand rungs of the β-ladder that forms the surface together with the β-roll. The unstructured and hydrophilic outer face of NS1 is characterized by the “spaghetti loop” (residues 219–272) of the β-ladder domain where most immunodominant and highly targeted regions have been mapped^11,22,23^. Notably, the first high-resolution cryoEM structures of DENV2 recombinant secreted NS1 (rsNS1) and in complex with 5E3 by Shu et al. showed that rsNS1 predominantly forms tetramers^24^. This challenged the longstanding view that secreted NS1 (sNS1) was a barrel-shaped hexamer with lipid cargos^25–27^. Furthermore, our preprint presented cryoEM structures of DENV2 infection-derived secreted NS1 (isNS1), which showed NS1 dimers complexed with HDL^28^ instead of the aforementioned barrel-shaped hexamers. This notion supports earlier reports that rsNS1 associates with HDL to trigger pro-inflammatory responses^29,30^ and uses scavenger receptor B1 (SRB1) as a cell receptor in cultured cells^31^. The observation that recombinantly derived flavivirus rsNS1 is primarily a tetramer, while its infection-derived physiologically relevant form, isNS1, is docked on HDL needs to be explored further.

To date there have been five published reports on antibody-NS1 complex structures. The Fab 22NS1 in complex with WNV C-terminal NS1 (NS1c; residues 172–352) bound to the loop face of the C-terminal β-ladder domain^32^. On the other hand Fab/ScFv 2B7 complex with DENV1/2 NS1^13^, Fab 1G5.3 complex with DENV2 NS1c (residues 174–352) or ZIKV NS1c (residues 272–339)^12^, and Fab 5E3 complex with DENV2 NS1^24^ showed a similar pattern of binding, albeit at different orientations at the distal end of the NS1 C-terminal β-ladder domain. The resolved structures of antibodies 2B7^13^, 1G5.3^12^, and 5E3^24^ bound to the NS1 β-ladder domain suggested that the protective effect through the Fcγ-independent pathway may be attributed to steric hindrance of the sNS1 interaction with lipid membranes.

To extend our understanding of how antibodies recognize and interact with ZIKV NS1, we determined the cryoEM structures of ZIKV rsNS1 alone and in complex with human monoclonal antibodies (AA12, EB9, and GB5), at 2.9 Å, 3.1 Å, 2.8 Å, and 3.8 Å, respectively. We observed tetramers of ZIKV rsNS1 (dimer of dimer), similar to the “loose” tetramers reported at 8.3 Å by Shu et al.^24^. In addition, ZIKV rsNS1 at a lower concentration forms a filamentous chain driven largely by the complementary surface charge interactions of the wing ladder. The three antibodies used in our study did not disrupt ZIKV NS1 tetramers into dimers. Compared to the available structures, our study presents a new subset of antibody-NS1 complex structures where the antibody binds between the wing and β-ladder domains of the NS1 protein, highlighting the presence of novel B cell epitopes in ZIKV NS1. Finally we also show that ZIKV isNS1 is found in complex with apoA1 as NS1 dimers docked onto HDL, similar to earlier reports for DENV2^28,30^.

## Results

### Recombinant secreted ZIKV NS1 forms asymmetric tetramers

**Fig. 1:**
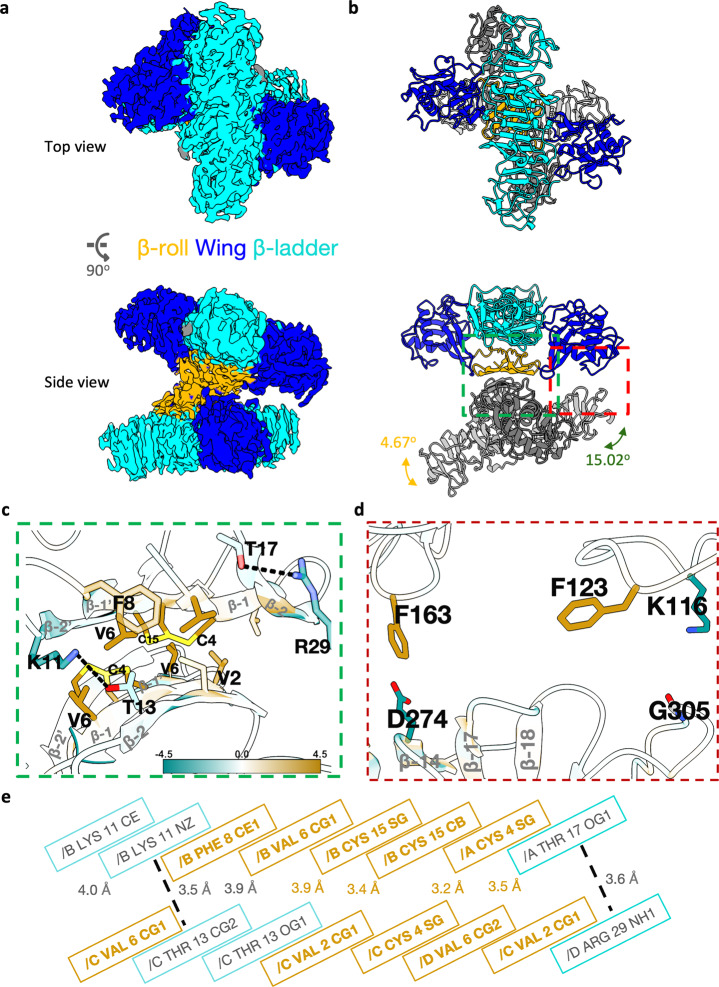
ZIKV MR766 rsNS1 cryoEM structure. **a** cryoEM density map with density threshold set at 0.3, colored near atoms of **b** the best fitted model with its three domains, being the β-roll (orange, residues 1–30), wing (blue, residues 31–179), and β-ladder (cyan, residues 180–352). The bottom dimer of NS1 in (**b**) is colored in gray (side and top views are as shown). Bi-directional arrows indicate the observed rotational angles of the dimer movement. Green and red dashed boxes in (**b**) outline the tetramer interface with close-up views of **c** the β-roll region and **d** between the wing and β-ladder of the dimers in the asymmetric tetramer. The key interacting residues are labeled with their single-letter codes and shown as sticks colored by heteroatom. NS1 is colored according to its lipophilicity potential (see key: a higher value corresponds to a higher level of hydrophobicity). The secondary structures are labeled as shown in gray. Measured distances are shown for (**d**). **e** Schematic representation of the NS1 dimer-dimer interactions observed at the β-roll interface. Hydrogen bonds are shown as dashed lines. Hydrophobic residues and the distances between them are colored in dark goldenrod as per color key in (**c**).

We expressed and purified full-length recombinant ZIKV NS1 (Uganda strain MR766; Zv sNS1_MR766wt_) with a carboxy (C)-terminus poly-histidine (HIS)-tag from the supernatant of transfected Expi293 cells. Unexpectedly, we observed that ZIKV rsNS1 forms filaments (Supplementary Fig. 1). Subsequent helical reconstruction of ZIKV rsNS1 filaments resulted in a low-resolution map of 8 Å, which could be fitted with ZIKV rsNS1 dimers. These ZIKV rsNS1 filaments are flexible and composed of asymmetric tetramers (dimer pairs) stacked along their exposed wing-ladder domain surfaces which possess complementary electrostatic surfaces (Supplementary Fig. 1). To achieve a higher resolution cryoEM structure of ZIKV rsNS1, we obtained a suitable dataset for single-particle analysis by increasing the sample concentration on graphene grids^33^. We similarly observed asymmetric tetramers and achieved an improved cryoEM map of 2.9 Å, although the distal ends of the β-ladder and wing domains are poorly resolved and there is significant smearing in one of the dimers (Fig. 1a and Supplementary Fig. 2). While focus refinements on the individual dimers did not improve the overall accuracy of the map, they showed obvious movement between them (Supplementary Fig. 2). We proceeded using the overall tetramer map to model all the residues, including uncertain regions such as the flexible loop at the wing tip, for completeness and interpretation of the protein movement. The range of motion hinges around the β-roll interface with movements up to 15.02° rotation (Fig. 1b, Supplementary Fig. 2, and Movie File 1). The dimer-dimer interface consists of the β-rolls oriented almost perpendicularly to each other (Fig. 1b, c) with potential interactions between Phe163 in the greasy finger of the wing domain and Asp274 of the β-ladder within 3.5 Å at the opposite protomer or other residues resulting in the assymetric tetramers (Fig. 1d and Supplementary Fig. 2). Only the β-roll interface is well resolved and the interacting residues are mapped in Fig. 1e.

### Comparison of recombinant secreted ZIKV NS1 asymmetric tetramers with prior CryoEM and crystal structures

**Fig. 2:**
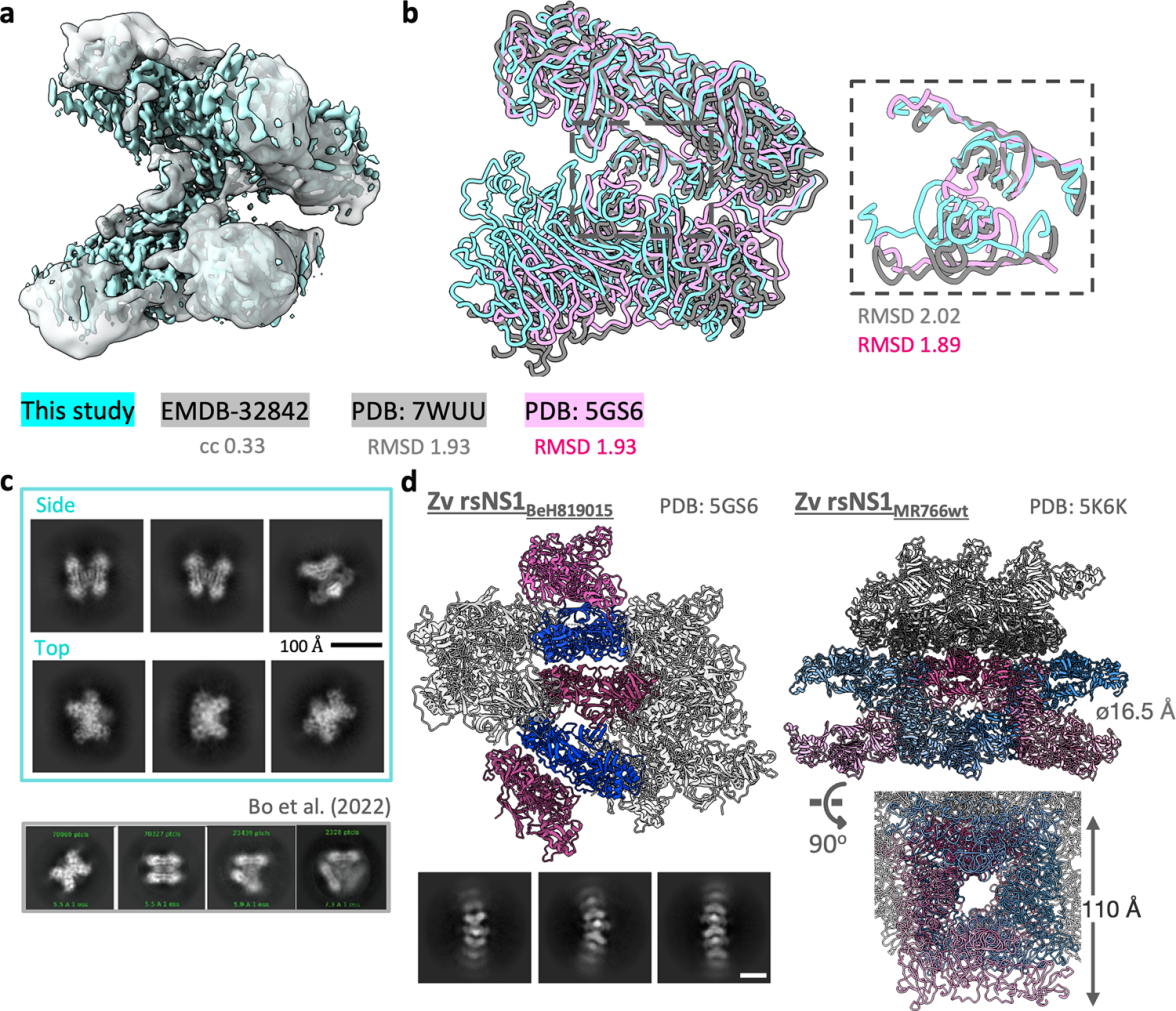
ZIKV MR766 rsNS1 cryoEM structure in comparison with Zv rsNS1 crystal and DENV2 rsNS1 CryoEM structures. **a** cryoEM map fitting of rsNS1 tetramer between ZIKV (2.9 Å, this study, in cyan) and DENV2 (8.3 Å, EMDB-32842, in gray), with densities thresholded at 0.3 and 4.66, respectively, have a calculated correlation of 0.33 determined using fitmap in ChimeraX, as labeled. **b** rsNS1 tetramer models from this study (in cyan) suposimposed to the DENV2 (PDB:7WUU, in gray) and ZIKV rsNS1 based on its crystal packing (PDB:5GS6, in pink). The tetramer interface at the β-roll is highlighted by the dashed gray box and the close-up view is shown on the right. The RMSD values are reported across all matched pairs as calculated using the matchmaker to a single chain for the overall model (left) and to the β-roll only (residues 1–30, right). **c** Representative 2D class averages of the ZIKV MR766 rsNS1 cryoEM dataset are shown in the cyan box, grouped by the side and top views. Black scale bar, 100 Å, as indicated. 2D class averages of the DENV rsNS1 cryoEM study from Fig. 1d of Bo et al. is reproduced herein (bottom) and boxed in gray for comparison. **d** The two reported crystal packing forms of the ZIKV rsNS1, from strains BeH819015 (PDB:5GS6, left) and MR766 (PDB:5K6K, right) are shown with their symmetry contacts displayed within 30 Å. These NS1 dimers form a single distinct filament-like (PDB:5GS6, left) or tubule-like (PDB:5K6K, right) complex as highlighted in shades of pink and blue, deduced based on their β-roll orientations. The other symmetry models are colored in gray. The length and internal diameter of a single structure were measured and labeled in the orientations presented (PDB:5K6K, right). We recapitulated ZIKV rsNS1 filament-like form as observed in our representative 2D class averages (bottom left, white scale bar, 100 Å) and cryoEM model (Supplementary Fig. 1).

Next, we compared our ZIKV MR766 rsNS1 tetramer CryoEM map with that of other structurally resolved DENV2 and ZIKV NS1 oligomers. We first superimposed our ZIKV NS1 tetramer map with the “loose” tetrameric rsNS1 of DENV2 PVP94/07 clinical strain (EMDB-32842)^24^ and observed that they largely overlapped (Fig. 2a). Next, we superimposed the corresponding model of the “loose” tetrameric rsNS1 CryoEM map of DENV2 PVP94/07 clinical strain (PDB:7WUU), and a previously reported crystal structure of ZIKV rsNS1 (strain: BeH819015 strain; PDB:5GS6), which could be tetrameric based on crystal packing (Fig. 2b)^8,24^. Overall, our tetrameric ZIKV rsNS1 is highly similar to the other reported structures (RMSD:1.93), although our model showed a different angle within the trajectory of the dimer scaffold (Fig. 2b and Supplementary Fig. 2). The representative 2D classes of ZIKV rsNS1 (Fig. 2c, top; and Supplementary Fig. 2) and DENV2 rsNS1 (Fig. 2c, bottom) reproduced from Shu et al.^24^ illustrate that only the “loose” tetramer form is observed in our study. Our ZIKV rsNS1 filament structure corroborates the ZIKV rsNS1 crystal contact form (PDB:5GS6) solved by Xu et al.^8^, as shown in Fig. 2d. Of note, further examination of the intermolecular contacts of the only other available ZIKV MR766 rsNS1 structure (PDB:5K6K)^21^, showed that ZIKV rsNS1 could also form a microtubule-like structure with a central hydrophobic cavity of ~16.5 Å.

### Characterization of human mAbs AA12, EB9, and GB5 against ZIKV NS1

**Fig. 3:**
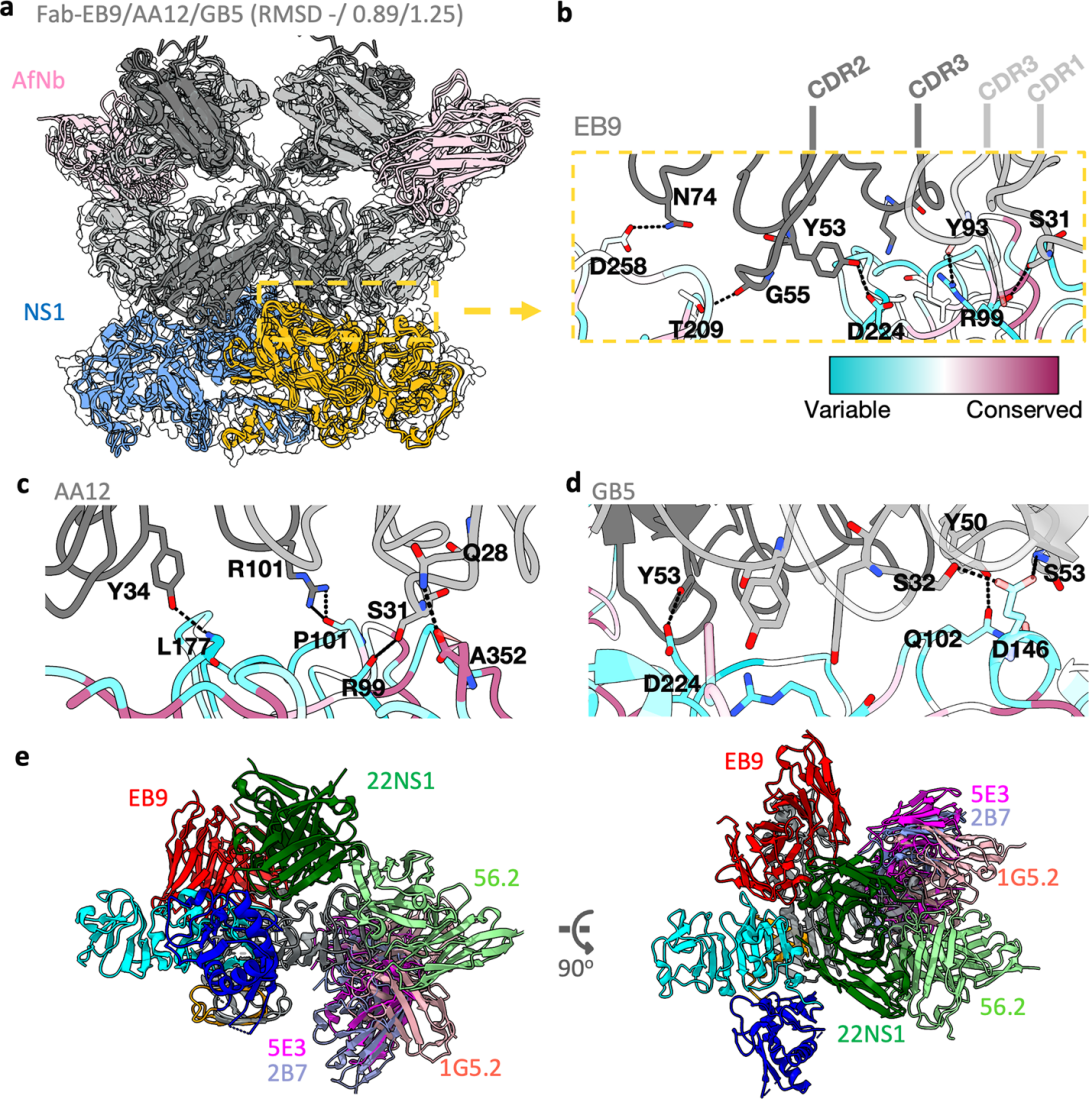
ZIKV MR766 rsNS1 in complex with human antibody fragments (Fab) EB9/AA12/GB5 and the antifab nanobody (AfNb). **a** Cartoon model of the complexes superimposed using matchmaker with respect to the EB9 complex. The RMSD value across all 352 paired atoms are as indicated, 0.89 and 1.25 for AA12 and GB5, respectively. AfNb is colored in pink, the Fab light and heavy chains are colored in dark and dim gray respectively, and the individual chains of the NS1 dimer is colored in cornflower blue and orange. Antibody-antigen binding interface is highlighted in an orange dashed box with details of the key interacting residue side chains shown for **b** EB9, **c** AA12, and **d** GB5, respectively, as sticks colored by heteroatom. The backbone is rendered as tubes, and only NS1 is colored according to residue conservation across flaviviruses (see key). The alignment is shown in Supplementary Fig. 6. Hydrogen bonds are shown as dashed lines and the complementarity-determining regions (CDRs) are labeled in (**b**). **e** EB9-NS1 model (red) superimposed with the solved antibody-NS1 structures at the NS1 β-ladder domain of 22NS1 (PDB:4OII, green), 56.2 (EMDB: 36483, light green), 1G5.2 (PDB:7BSC, in pink), 5E3 (PDB:7WUR, magenta), and 2B7 (PDB:6WER, light purple). The β-roll (orange), wing (blue) and β-ladder (cyan) domains of one of NS1 protomer are shown while the other one is depicted in gray for clarity of epitope regions. Only one copy of the Fab molecules and its variable light and heavy chains are shown.

The cryoEM maps of ZIKV rsNS1 in complex with human monoclonal antibodies (AA12, EB9, and GB5) were obtained at 3.1 Å, 2.8 Å, and 3.8 Å resolution, respectively (Supplementary Figs. 3–5). ZIKV rsNS1 was incubated with Fab AA12, EB9, or GB5. In addition, anti-Fab nanobody (AfNb), which binds to and increases the rigidity of the elbow linker between the variable and constant domains of the Fab LC, was added to aid in structure determination^34,35^. Both the heavy and light chains of Fabs AA12, EB9, and GB5 bind similarly to the surface of the ZIKV rsNS1 wing and β-ladder connector subdomain region with an observable weak density for AfNb (Fig. 3a). ZIKV rsNS1 remained tetrameric in all three cases, as seen in the 2D and 3D class averages, but the map density of one dimer was much weaker (Supplementary Figs. 3–5). This is indicative of a greater motion range of the dimer than ZIKV rsNS1 alone, as shown for the Fab EB9 data, with up to 28.2^o^ rotation observed (Supplementary Fig. 3). Fabs AA12 and EB9 datasets showed distinct classes of up to the theoretical maximum of four Fab molecules bound to the NS1 tetramer (Supplementary Figs. 3 and 4). Fab GB5 dataset only had up to two fab molecules bound to the exposed faces of the tetramer across all the class averages and with fewer particles picked (Supplementary Fig. 5). While the lower avidity observed for GB5 may be due to poorer sample quality or differences in data collection (Supplementary Table 1), it also correlates to being the weakest binder among the three^10^. To increase the map quality and determine the binding region, we first performed focused local refinements on the Fab domains bound to a ZIKV rsNS1 dimer (Supplementary Figs. 3–5). Comparing between masking strategies that either include or exclude the AfNb and Fab constant region did not lead to an obvious improvement in the overall map quality (Supplementary Fig. 3). Subtracting the poorly resolved dimer region made a bigger improvement that led to the final reported maps for AA12 and EB9 structures (Supplementary Figs. 3 and 4). For the Fab GB5 dataset, imposing a C2 symmetry during non-uniform refinement^36^ improved the map resolution from 4.25 to 3.76 Å based on the gold standard FSC curve cut-off at 0.143 and a corresponding improvement in the EMRinger^37^ score from 0.82 to 1.08 (Supplementary Fig. 5). The EMRinger Score assesses the goodness of fit between the model and the cryo-EM map, a value of 1.0 is typical for initial models of 3.2–3.5 Å resolution maps with increasing values expected for models of higher-resolution maps. We modeled all the residues, including uncertain regions such as the AfNb and the Fab constant regions, similar to what was done for ZIKV rsNS1 alone for structure completeness. The binding epitopes of Fab AA12, EB9, and GB5 were mapped to the wing (residues R99-R103, E146) and connector subdomains (residues D174–L177), which are generally poorly conserved sites (Fig. 3b, c, Supplementary Table 2, and Supplementary Fig. 6). Compared with the other antibody-NS1 complex structures obtained by superimposing the NS1 β-ladder domains, our structures represent a new subset of epitope regions compared to 22NS1^32^, 1G5.2^12^, 2B7^13^, 5E3^24^, and 56.2^28^ (Fig. 3e).

### ZIKV infection-derived NS1 from Vero cells is predominantly a dimer docked on high-density lipoproteins

**Fig. 4:**
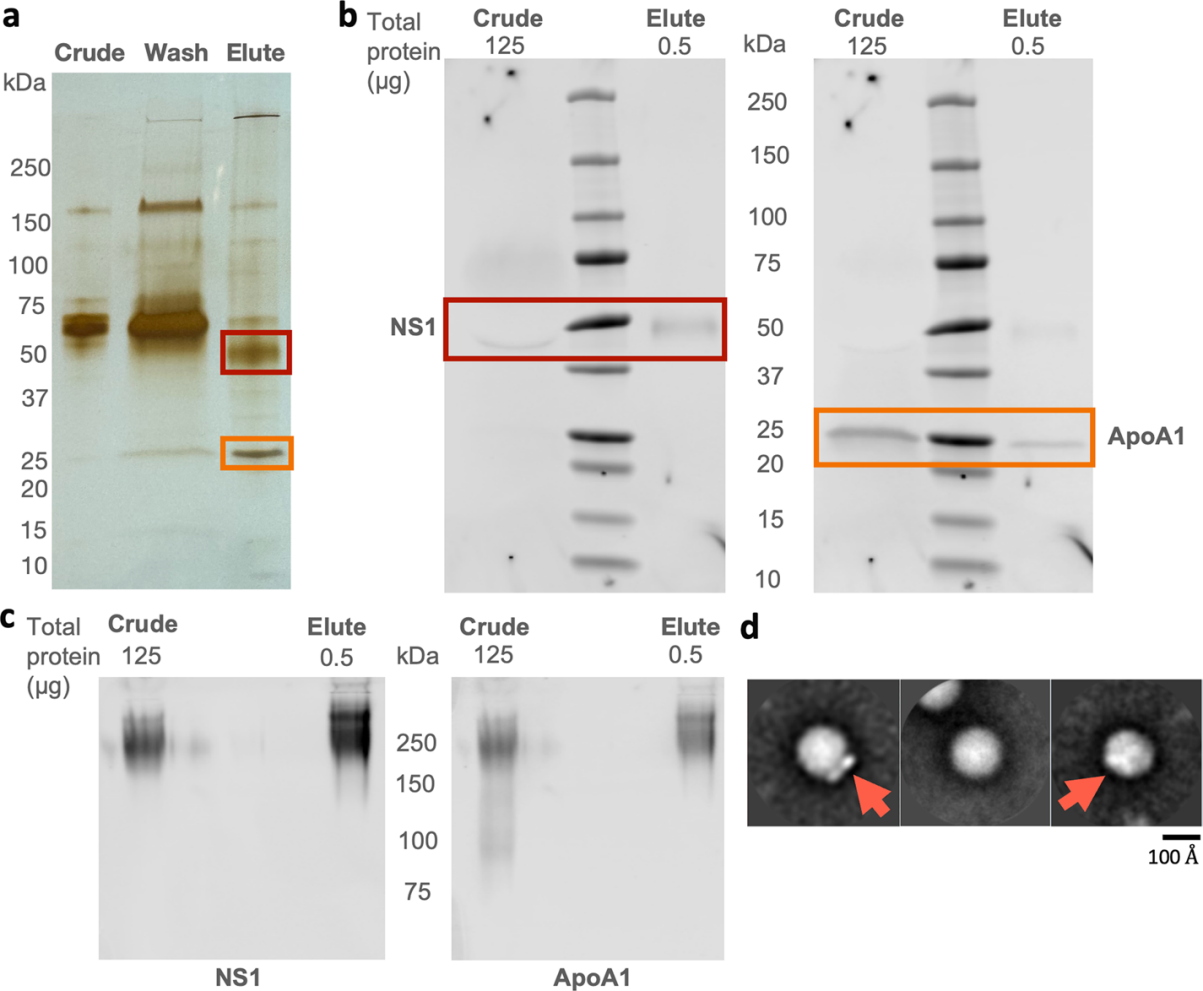
ZIKV MR766 sNS1 from the supernatant of infected Vero cells. **a** Silver-stained gel image of 100 ng of Crude and Elute immunoaffinity fractions that were separated on a 10% reducing SDS-PAGE gel. The maximum well volume (30uL; 0.06% of total volume) of the Wash immunoaffinity fraction was loaded, as the total protein concentration is below the Bradford assay detection limit. **b** Western blot validation of 50 kDa (boxed in red) and 25 kDa (boxed in orange) bands identified from (**a**) using an anti-ZIKV NS1 antibody (left; a gift from Yap Thai Leong, Experimental Drug Development Centre, A*STAR) and ApoA1 antibody (right; Biorbyt, orb240478) respectively. 125 µg and 500 ng total protein of Crude and Elute immunoaffinity fractions were separated on a 4–20% reducing SDS-PAGE gel. **c** Western blot detection of ZIKV NS1 (left) and ApoA1 (right) when 125 µg and 500 ng of total protein of Crude and Elute immunoaffinity fractions, respectively, were separated on a 10% Native-PAGE, using the same set of antibodies as described in (**b**). The full and unmodified images of gels and blots from (**a**–**c**) can be found in Supplementary Fig. 7. **d** Three representative 2D class averages derived from the negative electron micrographs of the Elute immunoaffinity fraction. Red arrows highlight the distinctive shape of the NS1 dimer protruding out of the spherical density as was demonstrated for DENV2 NS1 by Chew et al. and Benfrid et al.

Lastly, we also determined whether ZIKV sNS1 are predominantly found as dimers docked with HDL, similar to what was observed with DENV2 sNS1^28,30^. Using the same strategy as before^28^, we pulled down isNS1 with mAb EB9 and demonstrated that apoA1, a major component of HDL co-eluted with isNS1 (Fig. 4a–c). Negative-stain electron microscopy images of the eluted samples revealed distinctive wing-shaped protrusions of ZIKV NS1 dimers docked onto a spherical density that represents HDL in the 2D class averages (Fig. 4d). These representative 2D class averages are similar to those found for DENV2 sNS1^28,30^, providing more support for the notion that infection-derived flavivirus sNS1 incorporate HDL.

## Discussion

Prior biochemical and low resolution structural studies on DENV-1 sNS1 from infected Vero cells^26^ and recombinant DENV-2 sNS1 from baculovirus-infected Sf9 cells^27^ have shaped the longstanding view that sNS1 is a hexameric complex that can dissociate and bind to cellular membranes and contribute to cytopathic effects and disease^17^. However, recent works reported by Shu et al.^24^, Coelho et al.^30^, Benfrid et al.^30^, Alcala et al.^31^ and us^28^ tell a more nuanced and complicated story concerning the multimeric organization of sNS1. While a recent review^38^ acknowledged that recombinantly derived secreted DENV2 NS1 (rsNS1) are mostly tetramers^24^ that are able to bind to HDL^30^, it is still unclear how to reconcile the role of the different multimeric forms of secreted NS1, such as the hexameric NS1, in viral replication and disease pathogenesis. Here, we provide further evidence that the recombinant, secreted ZIKV NS1 forms tetramers with a distinct ability to oligomerize into filamentous structures (Figs. 1 and 2 and Supplementary Figs. 1 and 2). The direct relationship between the crystal contacts (PDB:5GS6)^8^ and the native multimeric ZIKV rsNS1 as have been observed under cryoEM (Fig. 2d, left) indicates the possibility that rsNS1 forms a microtubule-like structure observed in the crystal contacts of the other available ZIKV rsNS1 structures (PDB:5K6K; Fig. 2d, right)^21^. A bundle of two such ZIKV rsNS1 microtubles (~220 Å overall diameter) may explain the long and thin tubular protrusions extending out of liposomes observed by Ci et al. (2020) when ZIKV rsNS1 (strain: 1919) was added^39^. While the potential pathophysiological implications of rsNS1 filaments or microtubules are unclear and could simply be induced by the in vitro environment, such structures, as previously observed by us^28^ and others^24,30^, highlight discrepancies regarding the true soluble form of oligomeric NS1. There are clear structural differences among various sNS1 from different flaviviruses; recombinant NS1 from DENV2 and ZIKV (this study) are predominantly tetramers^24^ and infection-derived sNS1 of DENV2 form dimers bound to HDL^28^.

The three human mAb AA12, EB9, and GB5 studied were earlier shown by Bailey et al. to bind to Vero cells infected with either ZIKV MR766 or PRVABC59 strains but not to those cells when infected with DENV3^10^. Among them, GB5 was the weakest binder to MR766 by an order of magnitutde lower when compared to AA12 and EB9 (10^−7^ vs 10^−8^ nM range). AA12 and EB9 were then shown to have in vivo and Fcγ-dependent protection against lethal challenges of ZIKV MR766 and PRVABC59 strains in Stat2−/− mice. Similarly, the team later demonstrated that the ZIKV NS1-based vaccine is highly immunogenic and can elicit protective antibodies through Fc-dependent cell-mediated immunity^16^. Our antibody-NS1 complexes provide the structural basis by which monoclonal antibodies AA12, EB9, and GB5^10^ recognize epitopes on the surface of NS1 and provide in vivo protection in an Fcγ-dependent manner^10^. We found that unlike other antibodies such as 1G5.3^12^, 2B7^13^, 5E3^24^ and 56.2^28^, the antibodies AA12, EB9, and GB5^10^ do not interfere with the membrane interaction of NS1. Conservation analysis of antibody binding epitopes to the exposed charged surface of the wing and connector subdomain β-ladder helps to explain why such antibodies generally do not show cross-reactivity^10,11,23^ and are useful for flavivirus-specific rapid antigen assays^23^. Among the NS1 immunodominant regions^22^, it remains a keen interest to solve the structures of antibodies bound to the β-roll and connector subdomain of the wing region such as mAbs 3G2 and 4B8^14^. The membrane-associated face of NS1 may be exposed and unprotected when bound to antibodies such as 1G5.3^12^, 2B7^13^, 5E3^24^, or 56.2^28^ to allow antibodies to target the β-roll region.

In conclusion, our study highlights the polymorphic nature of rsNS1 as compared to the more physiologically relevant isNS1 and unravels the structural mechanistic basis for human mAb (AA12, EB9, and GB5)-mediated protection. The potential contribution of rsNS1 to biotherapeutic and vaccine development hinges on a structurally defined sample and key findings of our study provide important structural insights into NS1 alone and when bound to antibodies. Meanwhile, another preprint released by Pan et al.^40^ is showing cryoEM structures of ZIKV NS1 in complex with antibodies that are congruent with our conclusions.

## Methods

### Cells

Vero cells (Green African monkey kidney epithelial cells, ATCC) were cultured in DMEM containing 4.5 g/L glucose (Gibco) supplemented with 10% (v/v) fetal bovine serum (FBS) and 1% (v/v) penicillin-streptomycin (P/S), at 37 °C in 5% CO_2_. Expi293F and ExpiCHO cells (Thermo Fisher, USA) were cultured in serum-free Expi293 Expression Medium and FectoCHO CD medium (Polyplus-transfection S.A., France), respectively, at 37 °C in 8% CO_2_.

### Cloning, expression, and purification

Recombinant secreted NS1 ZIKV MR766 (Rhesus/1947/Uganda, GenBank accession no. MW143022.1) was cloned into mammalian secretion vector, pHL-Sec, with a hexahistidine tag at the C-terminus (NTU Protein Production Platform, Singapore). The plasmid was transfected into Expi293F cells using PEI Max (Polysciences, USA) following the manufacturer’s instruction and protein expression enhanced by sodium butyrate^41^. The supernatant containing recombinant secreted NS1 (rsNS1) was harvested on day 5, clarified by centrifuging at 4000 × *g* at 4 °C for 10 min and sterile filtered. The rsNS1 protein was purified by metal affinity chromatography using 1 mL of Ni-SMART Beads 6FF (Biobasic Asia Pacific, Singapore) in a gravity flow column pre-equlibrated in Buffer A1 (25 mM Tris, pH 8, 150 mM NaCl, 5% glycerol). rsNS1 was eluted with Buffer E (Buffer A1 supplemented with 400 mM NaCl, 500 mM imidazole) and further purified through gel-filtration chromatography (Superdex 200 10/300 GL column, Cytiva) in buffer A2 (25 mM Tris 8.0 and 150 mM NaCl). Collected protein fractions were concentrated up to 5 mg/mL using a 30 kDa molecular weight cutoff membrane concentrator. Protein purity was assessed by SDS-PAGE.

### Antibodies

Anti-Fab Nanobody (AfNb) coding sequence^35^ was synthesized and cloned into pET-28a between NcoI and XhoI sites with a hexahistidine tag at the C-terminus (Twist Bioscience, USA). The AfNb plasmid was expressed in the periplasm of competent BL23(DE3) Competent Cells (Thermo Fisher Scientific, USA) grown in Terrific Broth (HiMedia Laboratories, India) supplemented with 0.5% glycerol, 0.1% glucose, 0.2% lactose, and 1.25 mM MgSO_4_ for autoinduction^42^. AfNb was purified as described^43^ with additional dialysis step with Buffer W (50 mM Tris pH 7.5, 150 mM NaCl, 10 mM imidazole) prior to metal affinity chromatography using Ni-NTA Beads 6FF (Biobasic Asia Pacific, Singapore).

Recombinant full-length antibodies AA12/EB9/GB5 plasmids were obtained as described^10^ and site-directed mutagenesis at Cysteine residues 225, 229, and 226 to a stop codon at the respective heavy chain plasmids was performed to produce the Fab constructs. The plasmids were transfected into ExpiCHO cells (Thermo Fisher, USA) using FectoCHO® Expression system (Polyplus-transfection S.A., France) as per the manufacturer’s instructions. Secreted full-length antibodies in the supernatant were purified using Protein A beads (Genscript Biotech, Singapore) in a gravity flow column while Fab antibodies were purified using Protein L column on a AKTA purification system (Cytiva, USA). Both columns were pre-equilibrated in PBS, eluted using 0.1 M glycine pH 3.0, immediate neutralization with 1 M Tris-HCL pH 8.5, and buffer exchange to PBS using a molecular weight cutoff membrane concentrator.

### Analytical size exclusion chromatography

ZIKV rsNS1 was incubated with the AA12/EB9/GB5 antibody or its fragment and AfNb at a molar ratio of 1:2.1:2.2 for at least 1 h on ice. 50 µL of the respective complex was injected onto a Superdex 200 increase 3.2/300 GL column (GE Healthcare) connected to the µAKTA purification system (Cytiva, USA) buffer A2 (25 mM Tris 8.0 and 150 mM NaCl) at a constant flow rate of 0.06 mL/min. Chromatograms were analyzed on Unicorn 7. The purity and characterization of eluted fractions were determined using SDS-PAGE.

### Negative stain microscopy and data processing

Three µL of protein sample at concentration of 0.01 mg/mL were spotted on glow-discharged carbon grids, contrasted with 2% uranyl acetate, and imaged with a FEI Tecnai T12 microscope equipped with an Eagle 4-megapixel CCD camera (Thermo Fisher, USA). 26 micrographs were manually collected and processed using Scipion3^44^. CTF Estimation, manual and auto-picking were done using xmipp3^45^ resulting in a total of 5431 particles. This is followed by particle extraction and 2D classification using Relion^46^.

### Cryo-EM grid preparation and microscopy

QuantiFoil or UltrAuFoil R1.2/1.3 gold 300 mesh grid was used as is or covered with a graphene layer (Graphenea) following an adapted protocol^33^. The grids were glow-discharged for 10 s at low energy (Harrick Basic Plasma Cleaner) before use. 2.5 µL of protein sample at concentration of 0.35 mg/mL was applied to the grids, blotted for 3 s with blot force 1, and plunge-frozen in liquid ethane using Vitrobot (Thermo Fisher Scientific). Cryo-EM data collection parameters are summarized in Supplementary Table 1.

### Cryo-EM image single-particle processing

Collected movies were imported to Cryosparc v3.3 or later^47^. After Patch-Based Motion Correction and CTF Estimation, CTF-estimated maximum resolution better than 4.5 Å were selected. Particles were picked using a mix of Template Picker, Topaz^48^ and crYOLO v1.7.6^49^ and extracted at box sizes of 400, 256, 540, 600, 416 pixels for rsNS1 filament, rsNS1, rsNS1:AA12:AfNb, rsNS1:EB9:AfNb, and rsNS1:GB5:AfNb datasets, respectively. After two rounds of 2D classification with particles fourier cropped by four times and another two rounds of cleaning without downsampling, the particles were subjected to Ab initio Reconstruction. All resulting classes were refined respectively with no symmetry (C1) using Heterogeneous Refinement for further cleaning followed by rounds of Non-Uniform Refinement^36^, Local Refinement, and postprocessing using DeepEMhancer^50^. Rigid body motion of the protein is modeled using 3DFlex^51^. The details of the data collection and processing are summarized in the Supplementary Figs. 2–5 and Supplementary Table 1. The map for rsNS1:GB5:AfNb was further improved during Non-Uniform Refinement with C2 symmetry imposed.

### Model building and refinement

Initial map fitting was performed using predicted models generated using AlphaFold^52,53^, followed by manual corrections in Coot v0.9.8.x^54^ followed by cycles of real-space refinement in Phenix^55^ and ISOLDE^56^. Map fitting and figures representing the map and model features were done using UCSF ChimeraX^57^. As the map densities for AfNb and the constant domains of the fab are poor, this region was modeled based on one of the available CryoEM structures of AfNb-Fab (PDB:7PHP)^35^. The refinement statistics of the model are summarized in Supplementary Table 1.

### Cryo-EM image filament processing

The following data processing was performed in Relion v3.0.51^46^. A total of 3476 motion-corrected micrographs of NS1 were CTF-corrected using the CTFFIND program^58^. Filaments were manually picked using the manual picking job in Relion, resulting in a total of 343,520 helical segments. The box size used for extraction was 400, corresponding to a pixel size of 0.85 Å per pixel. After multiple rounds of 2D and 3D classifications, 167,332 particles were selected for further rounds of 3D classification. One of the two 3D classes was chosen, yielding in a total of 84,879 particles. Using these particles, 3D refinement was performed, resulting in a reconstruction at 8 Å resolution. The final reconstruction was imported into cisTEM^59^ for visualization purposes.

### Generation and purification of isNS1 from virus infection

Vero cells in 20 T175 flasks were infected at a multiplicity of 0.01 with ZIKV MR766 virus (GenBank accession: MW143022) for 1 h in serum-free DMEM and subsequently replaced with 25 mL of DMEM supplemented with 2% FBS per flask. The flasks were then incubated for 72 h at 37 °C in 5% CO_2_. The crude supernatant (500 mL) was then harvested, clarified, and filtered through a 0.2 μm filter membrane (Nalgene, Thermo Fisher, USA), followed by supplementation with 0.05% sodium azide and cOmplete EDTA-free protease inhibitor cocktail (Roche, Sigma-Aldrich). The crude supernatant was then concentrated 10-fold volume-wise using a Vivaflow 200 cassette with a 100 kDa MWCO (Sartorius, Germany) attached to a peristaltic pump (Cole-Parmer, USA). Five mL of Aminolink^TM^ resin (Thermo Fisher, USA) with immobilized antibody EB9 was then added to the crude supernatant and allowed end-over-end rotation overnight at 4^o^C for batch immunoaffinity purification. The slurry was then poured into a Econo-Pac® Chromatography Column (Bio-rad, USA), washed with filtered PBS (pH 7.4) for at least 10 column volumes and eluted with 3 column volumes of 0.1 M glycine (pH 2.7), immediately neutralized with 1 M Tris-HCl (pH 9.0). The eluted protein was dialyzed against PBS (pH 7.4) overnight at 4 °C and subsequently concentrated using a 100 kDa MWCO Amicon ultracentrifugal unit (Millipore, Merck, Germany). The total protein concentration was determined using Bradford assay (Bio-rad, USA). Protein quality was assessed on a 4–20% polyacrylamide SDS gel and a 10% Native polyacrylamide gel and transferred to PVDF membranes for western blot analysis against NS1 (gift from Yap Thai Leong, Experimental Drug Development Centre, A*STAR) and ApoA1 (Biorbyt, orb10643). Protein purity was assessed on a 4–20% polyacrylamide SDS gel and stained using Coomassie blue (0.2% Coomassie blue, 7.5% acetic acid, 50% methanol). The Precision Plus Protein Dual Color Standard (Bio-rad, USA) was used as the ladder for all protein gels in this work. Western blots and coomassie blue-stained gels were visualized with a Chemidoc Imager (Bio-rad, USA). The purified protein was stored at −80 °C until use.

## Supplementary information

Supplementary Movie1

Supplementary Movie2

Supplementary Materials

## Data Availability

The data that support this study are available from the corresponding authors upon reasonable request. The cryoEM maps and corresponding models have been deposited in the Electron Microscopy Data Bank (EMDB) and Protein Data Bank (PDB) under accession codes EMD-37676/PDB:8WO0 (ZIKV rsNS1 filament), EMD-37663/PDB:8WN8 (ZIKV rsNS1 tetramer), EMD-37670/PDB:8WNP (ZIKV rsNS1:FabAA12:AfNb), EMD-37678/PDB:8WO4 (ZIKV rsNS1:FabEB9:AfNb), and EMD-37673/PDB:8WNU (ZIKV rsNS1:FabGB5:AfNb).

## References

[1] Ruchusatsawat, K. et al. Long-term circulation of Zika virus in Thailand: an observational study. *Lancet Infect. Dis.* **19**, 439–446 (2019). 30826189 10.1016/S1473-3099(18)30718-7 PMC6511259

[2] Hill, S. C. et al. Emergence of the Asian lineage of Zika virus in Angola: an outbreak investigation. *Lancet Infect. Dis.* **19**, 1138–1147 (2019). 31559967 10.1016/S1473-3099(19)30293-2 PMC6892302

[3] Scaturro, P., Cortese, M., Chatel-Chaix, L., Fischl, W. & Bartenschlager, R. Dengue virus non-structural protein 1 modulates infectious particle production via interaction with the structural proteins. *PLoS Pathog.* **11**, e1005277 (2015). 26562291 10.1371/journal.ppat.1005277 PMC4643051

[4] Lindenbach, B. D. & Rice, C. M. Genetic interaction of flavivirus nonstructural proteins NS1 and NS4A as a determinant of replicase function. *J. Virol.* **73**, 4611–4621 (1999). 10233920 10.1128/jvi.73.6.4611-4621.1999 PMC112502

[5] Plaszczyca, A. et al. A novel interaction between dengue virus nonstructural protein 1 and the NS4A-2K-4B precursor is required for viral RNA replication but not for formation of the membranous replication organelle. *PLoS Pathog.* **15**, e1007736 (2019). 31071189 10.1371/journal.ppat.1007736 PMC6508626

[6] Noisakran, S. et al. Association of dengue virus NS1 protein with lipid rafts. *J. Gen. Virol.* **89**, 2492–2500 (2008). 18796718 10.1099/vir.0.83620-0

[7] Alcon-LePoder, S. et al. Secretion of flaviviral non-structural protein NS1: from diagnosis to pathogenesis. *Novartis Found. Symp.* **277**, 233–247 (2006). 17319166 10.1002/0470058005.ch17

[8] Xu, X. et al. Contribution of intertwined loop to membrane association revealed by Zika virus full-length NS1 structure. *EMBO J.* **35**, 2170–2178 (2016). 27578809 10.15252/embj.201695290 PMC5069551

[9] Puerta-Guardo, H. et al. Flavivirus NS1 triggers tissue-specific vascular endothelial dysfunction reflecting disease tropism. *Cell Rep.* **26**, 1598.e8–1613.e8 (2019). 30726741 10.1016/j.celrep.2019.01.036 PMC6934102

[10] Bailey, M. J. et al. Human antibodies targeting Zika virus NS1 provide protection against disease in a mouse model. *Nat. Commun.* **9**, 4560 (2018). 30385750 10.1038/s41467-018-07008-0 PMC6212565

[11] Wessel, A. W. et al. Antibodies targeting epitopes on the cell-surface form of NS1 protect against Zika virus infection during pregnancy. *Nat. Commun.* **11**, 5278 (2020). 33077712 10.1038/s41467-020-19096-y PMC7572419

[12] Modhiran, N. et al. A broadly protective antibody that targets the flavivirus NS1 protein. *Science* **371**, 190–194 (2021). 33414219 10.1126/science.abb9425

[13] Biering, S. B. et al. Structural basis for antibody inhibition of flavivirus NS1-triggered endothelial dysfunction. *Science* **371**, 194–200 (2021). 33414220 10.1126/science.abc0476 PMC8000976

[14] Yu, L. et al. Monoclonal antibodies against Zika virus NS1 protein confer protection via Fcγ receptor-dependent and -independent pathways. *mBio*10.1128/mBio.03179-20 (2021). 10.1128/mBio.03179-20 PMC7885117 33563822

[15] Chung, K. M. et al. Antibodies against West Nile Virus nonstructural protein NS1 prevent lethal infection through Fc gamma receptor-dependent and -independent mechanisms. *J. Virol.* **80**, 1340–1351 (2006). 16415011 10.1128/JVI.80.3.1340-1351.2006 PMC1346945

[16] Bailey, M. J. et al. Antibodies elicited by an NS1-based vaccine protect mice against Zika virus. *mBio*10.1128/mbio.02861-02818 (2019). 10.1128/mBio.02861-18 PMC6445944 30940710

[17] Glasner, D. R., Puerta-Guardo, H., Beatty, P. R. & Harris, E. The good, the bad, and the shocking: the multiple roles of dengue virus nonstructural protein 1 in protection and pathogenesis. *Annu. Rev. Virol.* **5**, 227–253 (2018). 30044715 10.1146/annurev-virology-101416-041848 PMC6311996

[18] Carpio, K. L. & Barrett, A. D. T. Flavivirus NS1 and its potential in vaccine development. *Vaccines*10.3390/vaccines9060622 (2021). 10.3390/vaccines9060622 PMC8229460 34207516

[19] van den Elsen, K., Chew, B. L. A., Ho, J. S. & Luo, D. Flavivirus nonstructural proteins and replication complexes as antiviral drug targets. *Curr. Opin Virol.* **59**, 101305 (2023). 36870091 10.1016/j.coviro.2023.101305 PMC10023477

[20] Akey, D. L. et al. Flavivirus NS1 structures reveal surfaces for associations with membranes and the immune system. *Science* **343**, 881–885 (2014). 24505133 10.1126/science.1247749 PMC4263348

[21] Brown, W. C. et al. Extended surface for membrane association in Zika virus NS1 structure. *Nat. Struct. Mol. Biol.* **23**, 865–867 (2016). 27455458 10.1038/nsmb.3268 PMC5951387

[22] Hertz, T. et al. Antibody epitopes identified in critical regions of dengue virus nonstructural 1 protein in mouse vaccination and natural human infections. *J. Immunol.* **198**, 4025–4035 (2017). 28381638 10.4049/jimmunol.1700029 PMC5603231

[23] Bosch, I. et al. Rapid antigen tests for dengue virus serotypes and Zika virus in patient serum. *Sci. Transl. Med.*10.1126/scitranslmed.aan1589 (2017). 10.1126/scitranslmed.aan1589 PMC6612058 28954927

[24] Shu, B. et al. CryoEM structures of the multimeric secreted NS1, a major factor for dengue hemorrhagic fever. *Nat. Commun.* **13**, 6756 (2022). 36347841 10.1038/s41467-022-34415-1 PMC9643530

[25] Flamand, M. et al. Dengue virus type 1 nonstructural glycoprotein NS1 is secreted from mammalian cells as a soluble hexamer in a glycosylation-dependent fashion. *J. Virol.* **73**, 6104–6110 (1999). 10364366 10.1128/jvi.73.7.6104-6110.1999 PMC112675

[26] Gutsche, I. et al. Secreted dengue virus nonstructural protein NS1 is an atypical barrel-shaped high-density lipoprotein. *Proc. Natl Acad. Sci. USA* **108**, 8003–8008 (2011). 21518917 10.1073/pnas.1017338108 PMC3093454

[27] Muller, D. A. et al. Structure of the dengue virus glycoprotein non-structural protein 1 by electron microscopy and single-particle analysis. *J. Gen. Virol.* **93**, 771–779 (2012). 22238236 10.1099/vir.0.039321-0

[28] Chew, B. L. A. et al. Secreted dengue virus NS1 from infection is predominantly dimeric and in complex with high-density lipoprotein. *eLife* **12**, RP90762 (2023). 10.7554/eLife.90762 PMC11126310 38787378

[29] Coelho, D. R. et al. ApoA1 neutralizes proinflammatory effects of dengue virus NS1 protein and modulates viral immune evasion. *J. Virol.* **95**, e0197420 (2021). 33827950 10.1128/JVI.01974-20 PMC8437349

[30] Benfrid, S. et al. Dengue virus NS1 protein conveys pro-inflammatory signals by docking onto high-density lipoproteins. *EMBO Rep.* **23**, e53600 (2022). 35607830 10.15252/embr.202153600 PMC10549233

[31] Alcala, A. C. et al. Dengue yirus NS1 uses scavenger receptor B1 as a cell receptor in cultured cells. *J. Virol.* **96**, e0166421 (2022). 34986002 10.1128/jvi.01664-21 PMC8906393

[32] Edeling, M. A., Diamond, M. S. & Fremont, D. H. Structural basis of Flavivirus NS1 assembly and antibody recognition. *Proc. Natl Acad. Sci. USA* **111**, 4285–4290 (2014). 24594604 10.1073/pnas.1322036111 PMC3964132

[33] Han, Y. et al. High-yield monolayer graphene grids for near-atomic resolution cryoelectron microscopy. *Proc. Natl. Acad. Sci. USA* **117**, 1009–1014 (2020). 31879346 10.1073/pnas.1919114117 PMC6969529

[34] Ereno-Orbea, J. et al. Structural basis of enhanced crystallizability induced by a molecular chaperone for antibody antigen-binding fragments. *J. Mol. Biol.* **430**, 322–336 (2018). 29277294 10.1016/j.jmb.2017.12.010

[35] Bloch, J. S. et al. Development of a universal nanobody-binding Fab module for fiducial-assisted cryo-EM studies of membrane proteins. *Proc. Natl Acad. Sci. USA* **118**, e2115435118 (2021). 34782475 10.1073/pnas.2115435118 PMC8617411

[36] Punjani, A., Zhang, H. & Fleet, D. J. Non-uniform refinement: adaptive regularization improves single-particle cryo-EM reconstruction. *Nat. Methods* **17**, 1214–1221 (2020). 33257830 10.1038/s41592-020-00990-8

[37] Barad, B. A. et al. EMRinger: side chain–directed model and map validation for 3D cryo-electron microscopy. *Nat. Methods* **12**, 943–946 (2015). 26280328 10.1038/nmeth.3541 PMC4589481

[38] Alcala, A. C. & Ludert, J. E. The dengue virus NS1 protein; new roles in pathogenesis due to similarities with and affinity for the high-density lipoprotein (HDL)? *PLoS Pathog.* **19**, e1011587 (2023). 37616216 10.1371/journal.ppat.1011587 PMC10449462

[39] Ci, Y. et al. Zika NS1-induced ER remodeling is essential for viral replication. *J. Cell Biol.*10.1083/jcb.201903062 (2020). 10.1083/jcb.201903062 PMC7041685 31868887

[40] Pan, Q. et al. Structural insights into the distinct protective mechanisms of human antibodies targeting ZIKV NS1. Preprint at *bioRxiv*10.1101/2023.10.16.562450 (2023).

[41] Gorman, C. M., Howard, B. H. & Reeves, R. Expression of recombinant plasmids in mammalian cells is enhanced by sodium butyrate. *Nucleic Acids Res.* **11**, 7631–7648 (1983). 6316266 10.1093/nar/11.21.7631 PMC326508

[42] Studier, F. W. In *Structural Genomics: General Applications* (ed. Chen, Y. W.) 17–32 (Humana Press, 2014).

[43] Pardon, E. et al. A general protocol for the generation of nanobodies for structural biology. *Nat. Protoc.* **9**, 674–693 (2014). 24577359 10.1038/nprot.2014.039 PMC4297639

[44] de la Rosa-Trevín, J. M. et al. Scipion: a software framework toward integration, reproducibility and validation in 3D electron microscopy. *J. Struct. Biol.* **195**, 93–99 (2016). 27108186 10.1016/j.jsb.2016.04.010

[45] de la Rosa-Trevín, J. M. et al. Xmipp 3.0: an improved software suite for image processing in electron microscopy. *J. Struct. Biol.* **184**, 321–328 (2013). 24075951 10.1016/j.jsb.2013.09.015

[46] Zivanov, J. et al. New tools for automated high-resolution cryo-EM structure determination in RELION-3. *Elife*10.7554/eLife.42166 (2018). 10.7554/eLife.42166 PMC6250425 30412051

[47] Punjani, A., Rubinstein, J. L., Fleet, D. J. & Brubaker, M. A. cryoSPARC: algorithms for rapid unsupervised cryo-EM structure determination. *Nat. Methods* **14**, 290–296 (2017). 28165473 10.1038/nmeth.4169

[48] Bepler, T. et al. Positive-unlabeled convolutional neural networks for particle picking in cryo-electron micrographs. *Nat. Methods* **16**, 1153–1160 (2019). 31591578 10.1038/s41592-019-0575-8 PMC6858545

[49] Wagner, T. et al. SPHIRE-crYOLO is a fast and accurate fully automated particle picker for cryo-EM. *Commun. Biol.* **2**, 218 (2019). 31240256 10.1038/s42003-019-0437-z PMC6584505

[50] Sanchez-Garcia, R. et al. DeepEMhancer: a deep learning solution for cryo-EM volume post-processing. *Commun. Biol.* **4**, 874 (2021). 34267316 10.1038/s42003-021-02399-1 PMC8282847

[51] Punjani, A. & Fleet, D. J. 3DFlex: determining structure and motion of flexible proteins from cryo-EM. *Nat. Methods* **20**, 860–870 (2023). 37169929 10.1038/s41592-023-01853-8 PMC10250194

[52] Jumper, J. et al. Highly accurate protein structure prediction with AlphaFold. *Nature* **596**, 583–589 (2021). 34265844 10.1038/s41586-021-03819-2 PMC8371605

[53] Mirdita, M. et al. ColabFold: making protein folding accessible to all. *Nat. Methods* **19**, 679–682 (2022). 35637307 10.1038/s41592-022-01488-1 PMC9184281

[54] Emsley, P., Lohkamp, B., Scott, W. G. & Cowtan, K. Features and development of Coot. *Acta Crystallogr. D Biol. Crystallogr.* **66**, 486–501 (2010). 20383002 10.1107/S0907444910007493 PMC2852313

[55] Liebschner, D. et al. Macromolecular structure determination using X-rays, neutrons and electrons: recent developments in Phenix. *Acta Crystallogr. D* **75**, 861–877 (2019). 10.1107/S2059798319011471 PMC6778852 31588918

[56] Croll, T. I. ISOLDE: a physically realistic environment for model building into low-resolution electron-density maps. *Acta Crystallogr. D Struct. Biol.* **74**, 519–530 (2018). 29872003 10.1107/S2059798318002425 PMC6096486

[57] Pettersen, E. F. et al. UCSF ChimeraX: structure visualization for researchers, educators, and developers. *Protein Sci.* **30**, 70–82 (2021). 32881101 10.1002/pro.3943 PMC7737788

[58] Rohou, A. & Grigorieff, N. CTFFIND4: fast and accurate defocus estimation from electron micrographs. *J. Struct. Biol.* **192**, 216–221 (2015). 26278980 10.1016/j.jsb.2015.08.008 PMC6760662

[59] Grant, T., Rohou, A. & Grigorieff, N. cisTEM, user-friendly software for single-particle image processing. *eLife* **7**, e35383 (2018). 29513216 10.7554/eLife.35383 PMC5854467

