# Supplementary figures and images for: Structural basis of Zika virus NS1 multimerization and human antibody recognition
Source: Npj Viruses. 2024 Apr 25;2:14. doi: 10.1038/s44298-024-00024-6 (PMC11721437; doi:10.1038/s44298-024-00024-6)

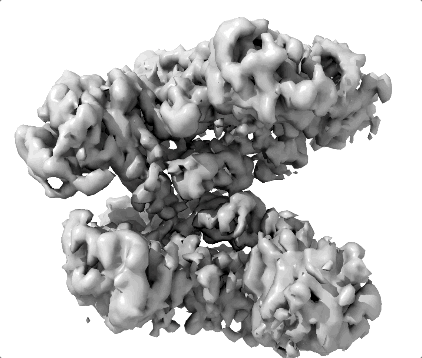

Supplement: Supplementary file 1 — Supplementary Movie1 [file 44298_2024_24_MOESM1_ESM.gif]

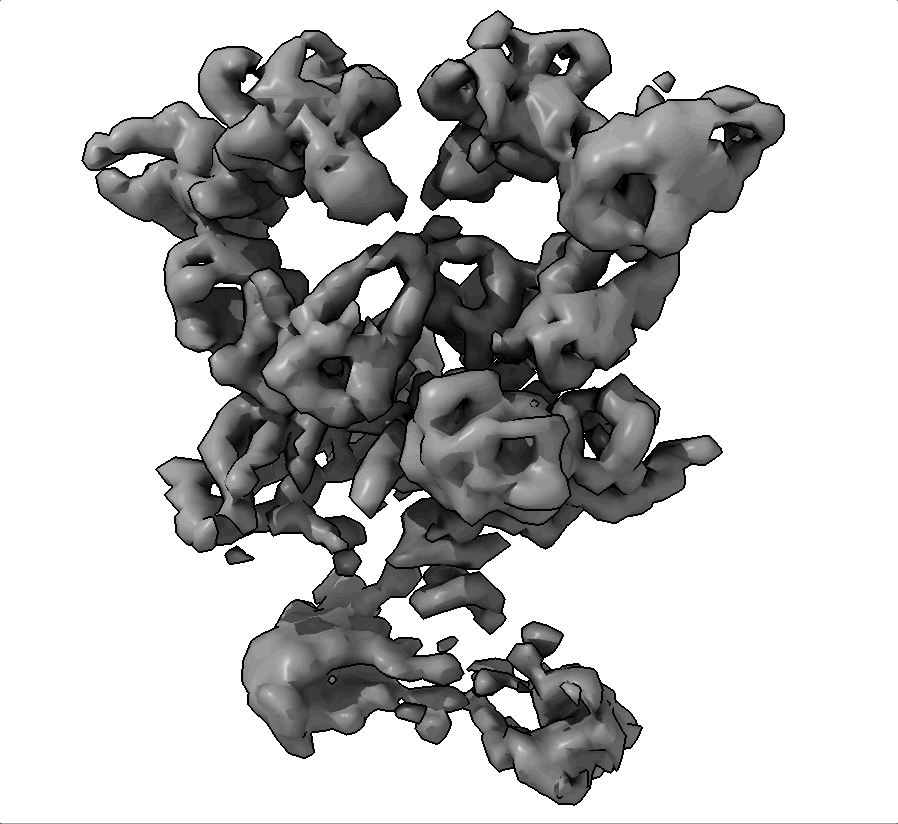

Supplement: Supplementary file 2 — Supplementary Movie2 [file 44298_2024_24_MOESM2_ESM.gif]
