# Supplementary material for: Structural basis of Zika virus NS1 multimerization and human antibody recognition
Source: Npj Viruses. 2024 Apr 25;2:14. doi: 10.1038/s44298-024-00024-6 (PMC11721437; doi:10.1038/s44298-024-00024-6)
Supplement: Supplementary file 3 — Supplementary Materials [file 44298_2024_24_MOESM3_ESM.docx]

Supplementary Materials for

**Structural basis of Zika virus NS1 multimerization and human antibody recognition**

**This PDF file includes:**

Supplementary Fig. 1 to 7

Supplementary Table 1 - 2

**Supplementary figures**

**
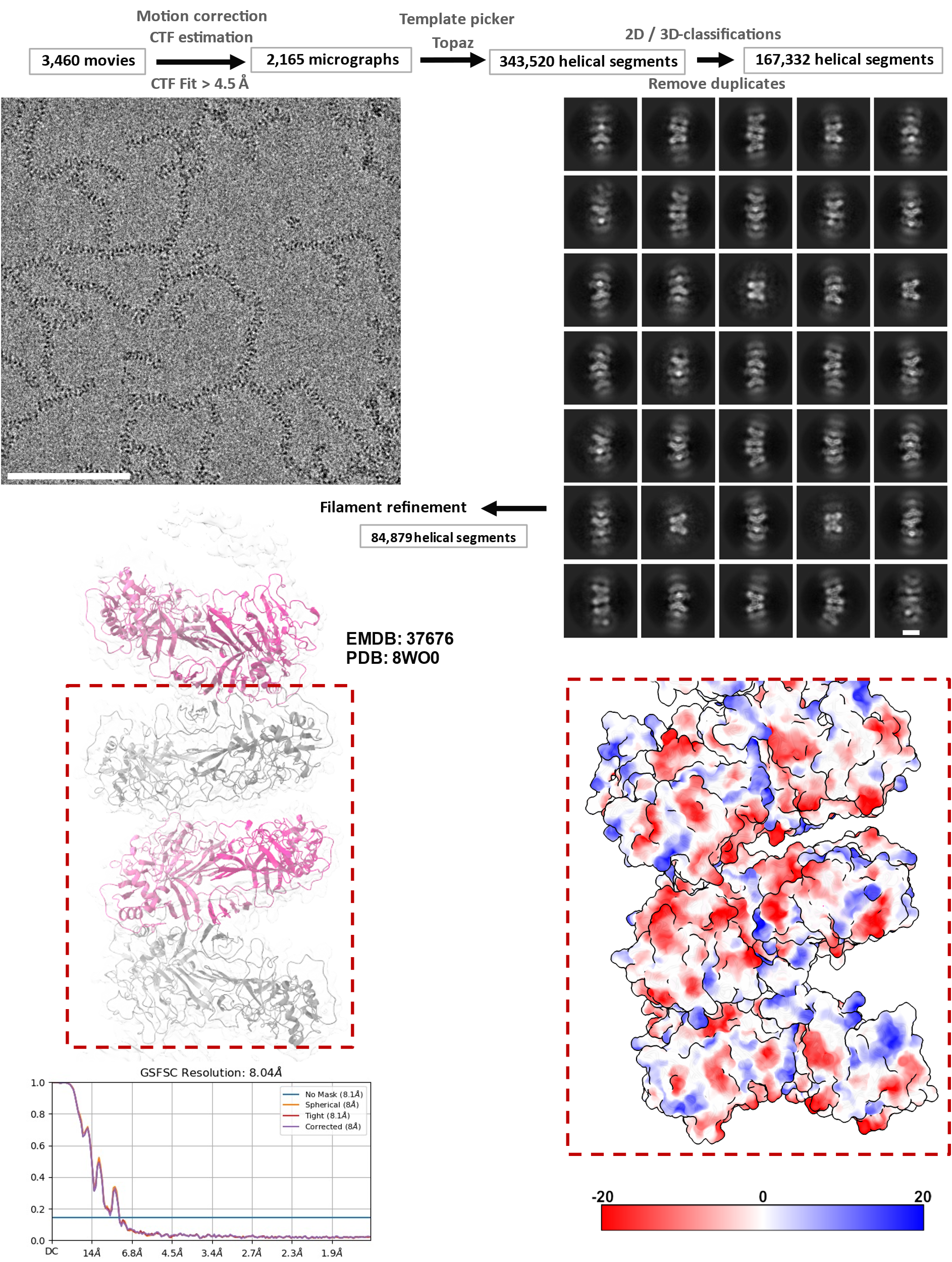
**

**Supplementary Figure 1. CryoEM reconstruction workflow for ZIKV MR766 rsNS1 filament.** Data analysis workflow and the corresponding number of images and particles are as shown, along with a representative motion-corrected micrograph with a white scale bar, 100 nm, and the resulting 2D classes of the picked particles with a white scale bar, 100 Å, are as shown. 3D model-map fitting, map is outlined in the background while the dimer models are colored alternating in magenta and grey. Red dashed box to highlight a close-up view of the model surface recolored by the electrostatic potential with the color key as shown. The FSC chart was generated in crysosparc4.0.


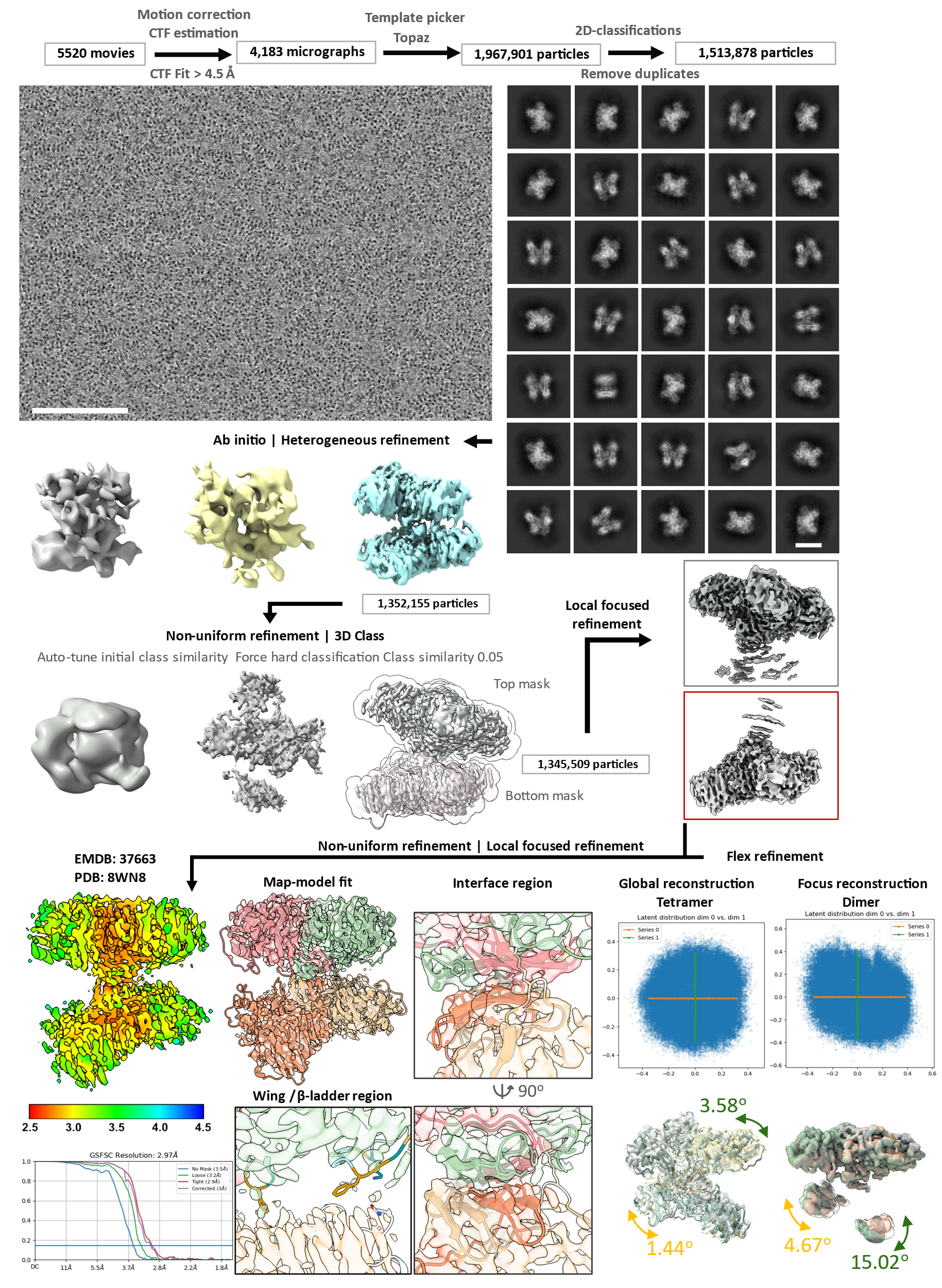


**Supplementary Figure 2. CryoEM reconstruction workflow for ZIKV MR766 rsNS1.** Data analysis workflow and the corresponding number of images and particles are as shown, along with a representative motion-corrected micrograph with a white scale bar, 100 nm, and the resulting 2D classes of the picked particles with a white scale bar, 100 Å. The 3D classes generated from ab initio to refinement stages are as depicted. Mask volumes were outlined in the background of the selected map in semi-transparent grey (top mask) or red (bottom mask), as labelled, for separate focused refinements of the dimers within the tetramer. One final cryoEM map volume was colored to resolution. Another final cryoEM map volume presents the overall model fitting and colored by chains, with zoom in panels of the β-roll interface and the wing/β-ladder region. The FSC curves was generated in crysosparc4.0. Flex refinement latent distribute charts based on either the overall or focused refined 3D maps were generated in crysosparc4.1. The corresponding rotational angles of the map-model movement in series 0 (orange) and series 1 (green) directions were visualized and measured using chimeraX. Refer to the Movie 1 file for the modelled movement of the ZIKV sNS1.


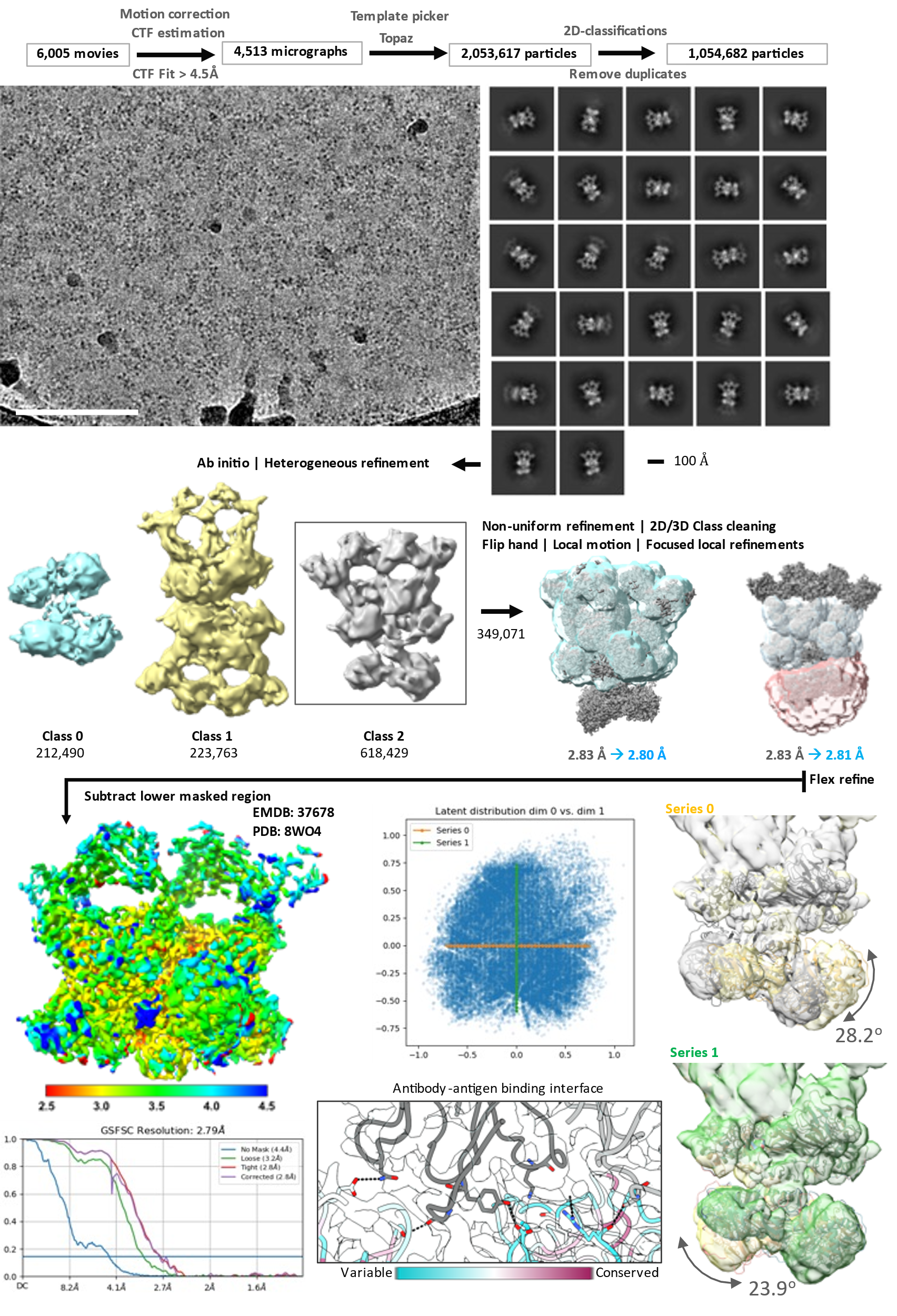


**Supplementary Figure 3. CryoEM reconstruction workflow for ZIKV MR766 rsNS1 incubated with Fab EB9 and anti-fab nanobody (AfNb).** Data analysis workflow and the corresponding number of images and particles are as shown, along with a representative motion-corrected micrograph with a white scale bar, 100 nm, and the resulting 2D classes of the picked particles with a black scale bar, 100 Å. The 3D classes generated from ab initio to refinement stages are as depicted with their particle numbers. Semi-transparent mask volumes were outlined and colored by different regions of the selected map for separate focused refinements of the antibody-dimer complex (cyan and blue) or for signal subtraction (red) before further refinement. The final map volume was colored to resolution and the corresponding FSC curves displayed below was generated in crysosparc4.0. The quality of the map-model fitting at the antibody-NS1 binding interface is as shown, with the heavy chain colored in grey, light chain in dark grey, the NS1 colored by the sequence conservation, and the map is transparent with silhouette. Flex refinement latent distribute charts based on the overall refined 3D map prior to signal subtraction were generated in crysosparc4.1. The corresponding rotational angles shown of the map-model movement in series 0 (orange) and series 1 (green) directions were visualized and measured using chimeraX. Refer to the Movie 2 file for the modelled movement of the ZIKV sNS1 in complex with Fab EB9 and AfNb.


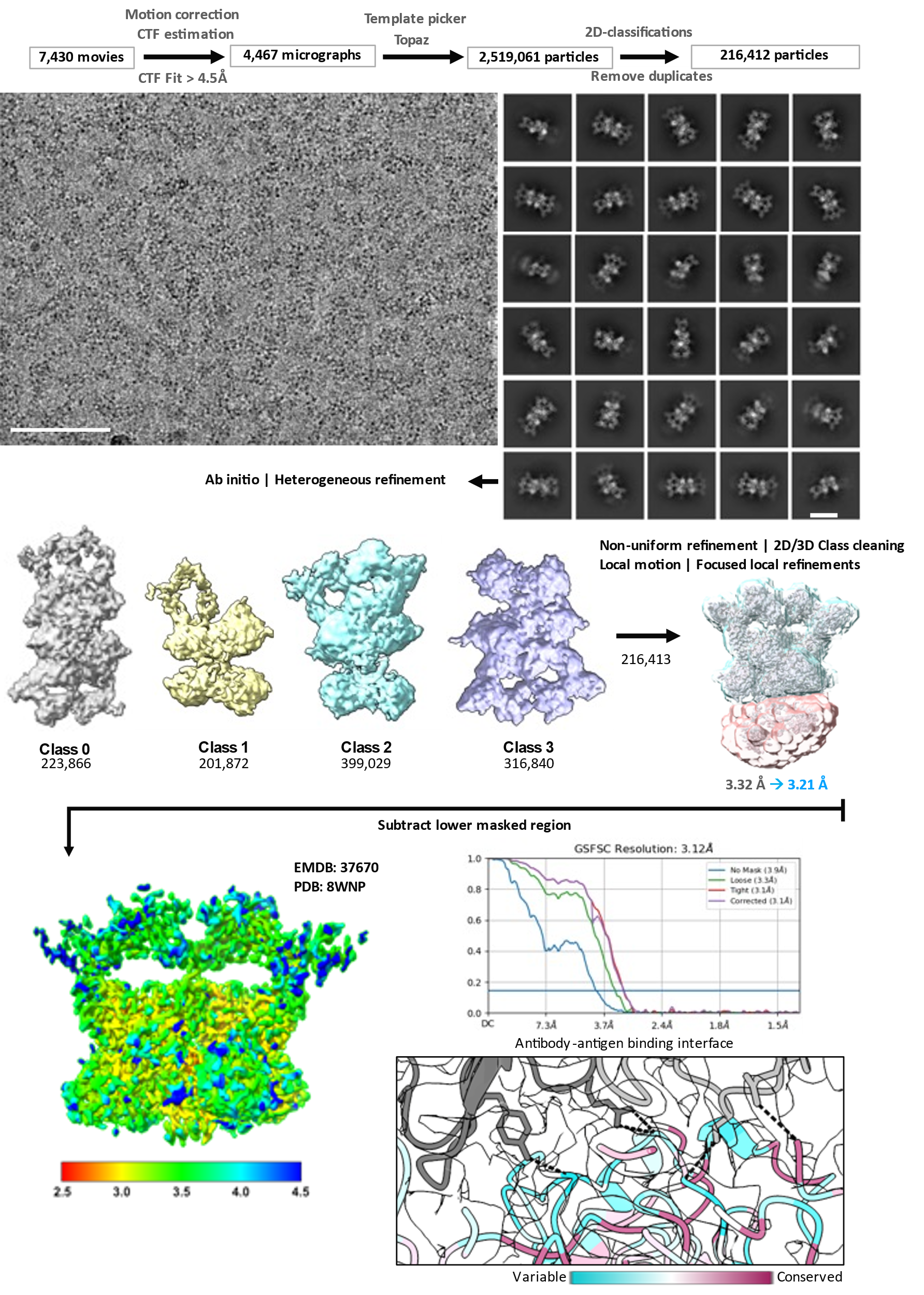


**Supplementary Figure 4. CryoEM reconstruction workflow for ZIKV MR766 rsNS1 incubated with Fab AA12 and anti-fab nanobody (AfNb).** Data analysis workflow and the corresponding number of images and particles are as shown, along with a representative motion-corrected micrograph with a white scale bar, 100 nm, and the resulting 2D classes of the picked particles with a white scale bar, 100 Å. The 3D classes generated from ab initio to refinement stages are as depicted with their particle numbers. Semi-transparent mask volumes were outlined and colored by different regions of the selected map for separate focused refinements (cyan) of the antibody-dimer complex or for signal subtraction (red) before further refinement. The final map volume was colored to resolution and displayed with its FSC curves as generated in crysosparc4.0. The quality of the map-model fitting at the antibody-NS1 binding interface is as shown, with the heavy chain colored in grey, light chain in dark grey, the NS1 colored by the sequence conservation, and the map is transparent with silhouette.


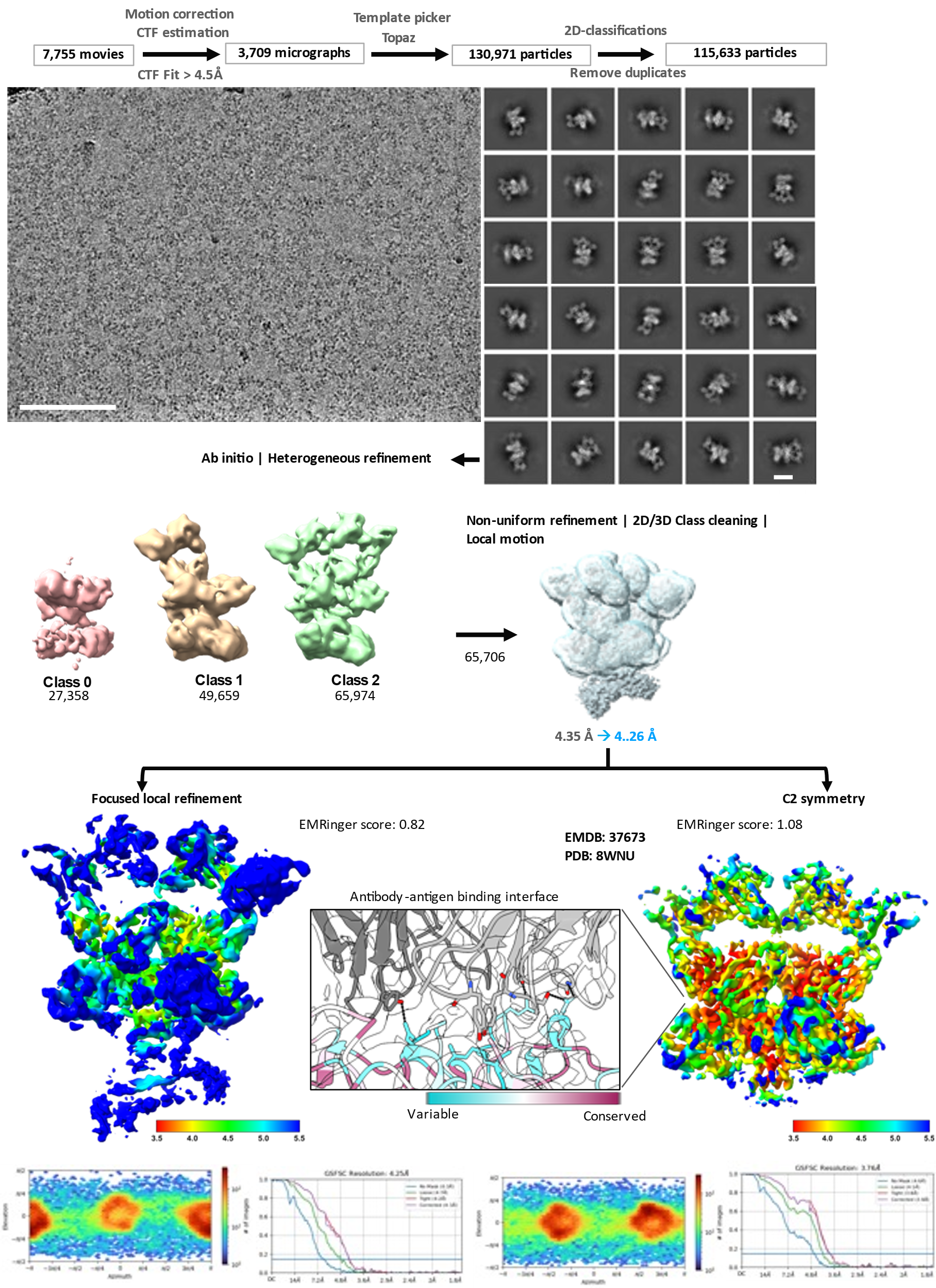


**Supplementary Figure 5.** CryoEM reconstruction workflow for ZIKV MR766 rsNS1 incubated with Fab GB5 and anti-fab nanobody (AfNb). Data analysis workflow and the corresponding number of images and particles are as shown, along with a representative motion-corrected micrograph with a white scale bar, 100 nm, and the resulting 2D classes of the picked particles with a white scale bar, 100 Å. The 3D classes generated from ab initio to refinement stages are as depicted with their particle numbers. Semi-transparent mask volume was outlined and colored in cyan of the selected map for focused local refinement with or without C2 symmetry applied. The final map volume was colored to resolution, and the FSC curves and the heat map of the viewing direction distribution as depicted were generated in crysosparc4.0. The quality of the map-model fitting at the antibody-NS1 binding interface is as shown, with the heavy chain colored in grey, light chain in dark grey, the NS1 colored by the sequence conservation, and the map is transparent with silhouette.


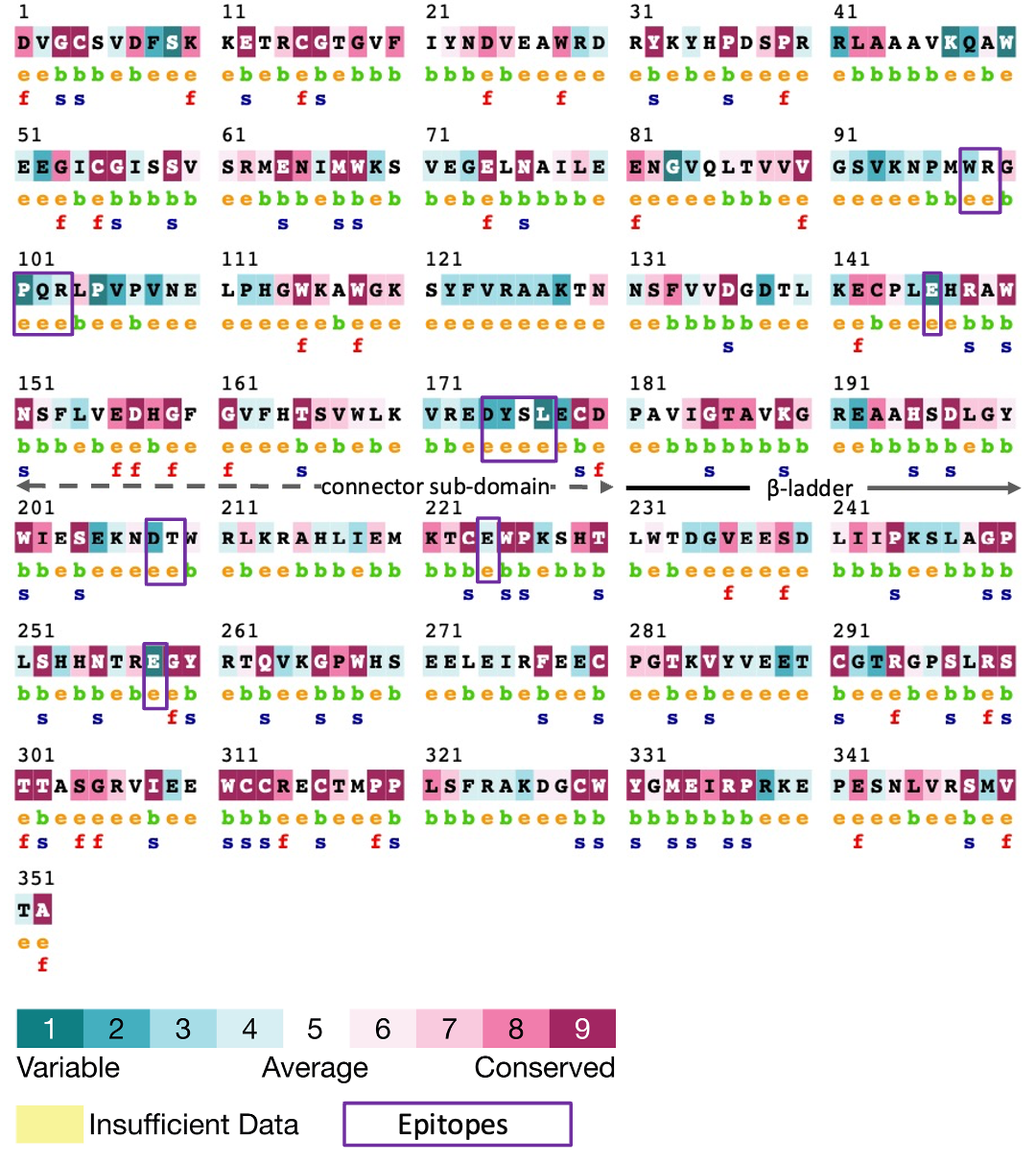


**Supplementary Figure 6.** Conservation quality of ZIKV NS1 amino acid residues based on ConSurf’s color grades (1-9) as shown in the legend. The distribution of structural and functional residues over the structure according to ConSurf neural-network algorithm[^24^](#_ENREF_24), 'e' indicates an exposed residue, 'b' that of buried residue, 'f' indicates predicted functional residue (highly conserved and exposed), and 's' indicates predicted structural residue (highly conserved and buried). The connector sub-domain between the wing domain to the β-ladder (151-180) is indicated with a bidirectional dashed arrow, and a forward solid arrow to indicate the start of β-ladder domain. The target epitope of the anti-zikv human antibodies studied are mapped out in purple line boxes. Sequence similarity calculated using Clustal Omega v1.2.4[^25^](#_ENREF_25).


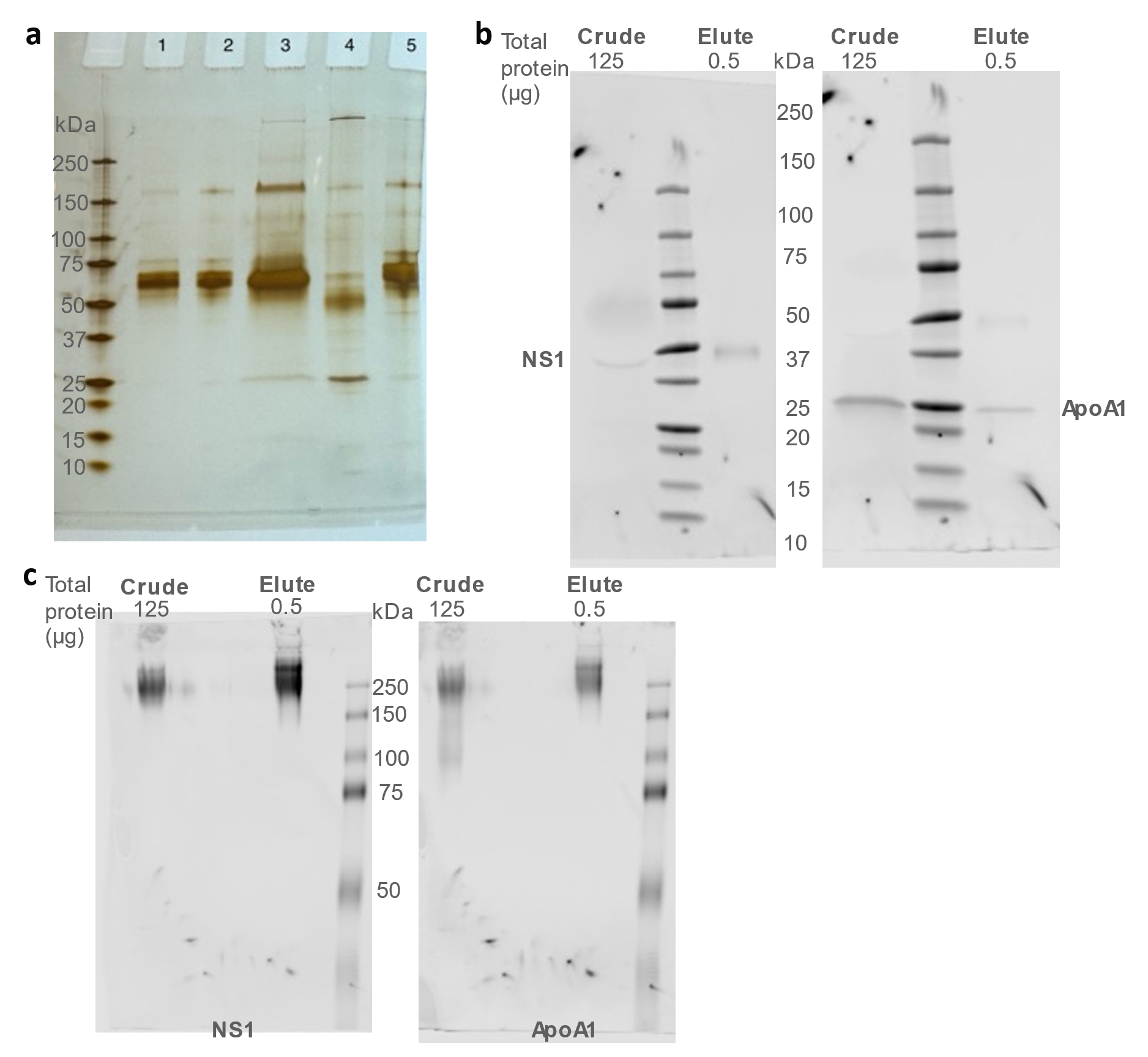


**Supp. Fig. 7. Full and unmodified images of gels and blots for ZIKV MR766 sNS1 from the supernatant of infected Vero cells presented in Figure 4. (a)** Silver-stained gel image of 100 ng of Crude and Elute immunoaffinity fractions that were separated on a 10% reducing SDS-PAGE gel. The maximum well volume (30uL; 0.06% of total volume) of the Wash immunoaffinity fraction was loaded, as the total protein concentration is below the Bradford assay detection limit. **(b)** Western Blot validation of 50 and 25 kDa bands identified from (a) using an anti-ZIKV NS1 antibody (left; a gift from Yap Thai Leong, Experimental Drug Development Centre, A*STAR) and ApoA1 antibody (right; Biorbyt, orb240478) respectively. The total protein of Crude and Elute immunoaffinity fractions loaded on a 4-20% reducing SDS-PAGE gel are as labelled. **(c)** Western Blot detection of ZIKV NS1 (left) and ApoA1 (right) when 125 µg and 500 ng of total protein of Crude and Elute immunoaffinity fractions respectively, were separated on a 10% Native-PAGE, using the same set of antibodies as described in (b).

| **Supplementary table 1. Cryo-EM data collection, processing, and validation statistics** | | | | |  |
| --- | --- | --- | --- | --- | --- |
| **ZIKV MR766wt**  Samples | **sNS1**  **Filament** | **sNS1** | **sNS1:AA12** | **sNS1:EB9** | **sNS1**:**GB5** |
| EMDB | 37676 | 37663 | 37670 | 37678 | 37673 |
| PDB | 8WO0 | 8WN8 | 8WNP | 8WO4 | 8WNU |
| Grid type | AuFoil | gAuFoil | gAuFoil | gAuFoil | gQtFoil-Au |
| Microscope | Titan Krios G3 | | | |  |
| Voltage (keV) | 300 | | | |  |
| Camera, Gatan | K2 | K3 | K3 | K3 | K3 |
| Nominal magnification (kx) | 120 | 105 | 130 | 130 | 105 |
| Pixel size (Å/pixel) | 0.85 | 0.86 | 0.68 | 0.68 | 0.86 |
| Total electron dose (e-/Å^2^) | 48 | 50 | | |  |
| Exposure rate (e-/ Å^2^/s) | 1.2 | 1 | | |  |
| Number of frames | 40 | 50 | | |  |
| Defocus range (μm) | -0.5 to -2.0 | | | |  |
| Automation software | EPU | | | |  |
| Energy filter slit width | 20 | | | |  |
| Micrographs collected | 3460 | 5520 | 7430 | 6005 | 7755 |
| Micrographs post-clean | 2165 | 4183 | 4467 | 4513 | 3709 |
| Initial number of particles | 343,520 | 1,967,901 | 2,519,061 | 2,053,617 | 130,971 |
| Final number of particles | 84,879 | 1,345,509 | 216,412 | 349,070 | 65,706 |
| Map resolution | 8 | 2.9 | 3.1 | 2.8 | 3.8 |
| Symmetry imposed | C1 | C1 | C1 | C1 | C2 |
| Helical rise (Å) | 91 | - | | |  |
| Helical rotation (°) | 186 | - | | |  |
| FSC threshold | 0.143 | | | |  |
| **Refinement** |  |  |  |  |  |
| phenix.real_space_refineresolution cutoff | 8.0 | 3.0 | 3.3 | 3.0 | 4.0 |
| Resolution Estimates (Å) unmasked and masked reconstructions at FSC (0.143 / 0.5) | 7.5/4.7  7.3/17.4 | 2.9/3.3  3.1/3.4 | 3.1/3.7  3.1/3.5 | 2.8/3.6  2.8/3.4 | 3.8/4.5  3.8/4.4 |
| Map sharpening B factor (Å^2^) | -200 | -135.2 | -68.6 | -53.8 | -108.9 |
| Model composition |  |  |  |  |  |
| Non-hydrogen atoms | 50110 | 22180 | 27809 | 27891 | 27823 |
| Protein residues | 3520 | 1408 | 1822 | 1830 | 1824 |
| Ligands | NAG: 8 | NAG: 2 | NAG: 2 | NAG: 2 | NAG: 2 |
| Global CC (CCvol) | 0.63 | 0.72 | 0.62 | 0.60 | 0.62 |
| Local CC (CCmask) | 0.69 | 0.75 | 0.65 | 0.64 | 0.64 |
| *B* factors (Å^2^) |  |  |  |  |  |
| Protein | 779.67 | 95.34 | 64.69 | 46.84 | 88.59 |
| Ligand | 531.96 | 88.05 | 84.51 | 71.29 | 74.83 |
| R.m.s. deviations |  |  |  |  |  |
| Bond lengths (Å) | 0.003 | 0.004 | 0.003 | 0.002 | 0.002 |
| Bond angles (°) | 0.598 | 0.642 | 0.637 | 0.528 | 0.579 |
| Validation |  |  |  |  |  |
| MolProbity score | 2.40 | 2.27 | 1.83 | 1.57 | 2.01 |
| Clash score | 28.54 | 9.78 | 1.83 | 5.70 | 9.85 |
| Poor rotamers (%) | 0.00 | 2.37 | 0.32 | 0.96 | 0.13 |
| Ramachandran plot |  |  |  |  |  |
| Favored (%) | 92.66 | 92.29 | 92.83 | 96.19 | 91.83 |
| Allowed (%) | 7.31 | 7.71 | 7.06 | 3.81 | 7.94 |
| Disallowed (%) | 0.03 | 0 | 0.11 | 0.0 | 0.22 |
| CaBLAM outliers (%) | 3.85 | 3.52 | 3.67 | 1.65 | 3.75 |
| EMRinger score | - | 2.28 | 1.74 | 2.24 | 1.09 |

| **Supplementary table 2. Zv rsNS1MR766wt epitopes and contacts with AA12, EB9, GB5** | | | |
| --- | --- | --- | --- |
| **NS1 epitopes** | **AA12** | **EB9** | **GB5** |
| R99 | (VL) Y93, S31 | (VL) S31 |  |
| P101 |  | (VH) 101 |  |
| Q102 |  |  | (VL) Y50 |
| E146 |  |  | (VL) S32, S53 |
| L177 |  | (VH) Y34 |  |
| T209 | (VH) G55 |  |  |
| D224 | (VH) Y53 |  | (VH) Y53 |
| D258 | (VH) N74 |  |  |
| A352 |  | (VL) Q28 |  |

**References**

1 Gorman, C. M., Howard, B. H. & Reeves, R. Expression of recombinant plasmids in mammalian cells is enhanced by sodium butyrate. *Nucleic Acids Res* **11**, 7631-7648 (1983). https://doi.org:10.1093/nar/11.21.7631

2 Bloch, J. S. *et al.* Development of a universal nanobody-binding Fab module for fiducial-assisted cryo-EM studies of membrane proteins. *Proceedings of the National Academy of Sciences* **118**, e2115435118 (2021). https://doi.org:doi:10.1073/pnas.2115435118

3 Studier, F. W. in *Structural Genomics: General Applications* (ed Yu Wai Chen) 17-32 (Humana Press, 2014).

4 Pardon, E. *et al.* A general protocol for the generation of Nanobodies for structural biology. *Nature Protocols* **9**, 674-693 (2014). https://doi.org:10.1038/nprot.2014.039

5 Bailey, M. J. *et al.* Human antibodies targeting Zika virus NS1 provide protection against disease in a mouse model. *Nat Commun* **9**, 4560 (2018). https://doi.org:10.1038/s41467-018-07008-0

6 de la Rosa-Trevín, J. M. *et al.* Scipion: A software framework toward integration, reproducibility and validation in 3D electron microscopy. *Journal of Structural Biology* **195**, 93-99 (2016). https://doi.org:https://doi.org/10.1016/j.jsb.2016.04.010

7 de la Rosa-Trevín, J. M. *et al.* Xmipp 3.0: An improved software suite for image processing in electron microscopy. *Journal of Structural Biology* **184**, 321-328 (2013). https://doi.org:https://doi.org/10.1016/j.jsb.2013.09.015

8 Zivanov, J. *et al.* New tools for automated high-resolution cryo-EM structure determination in RELION-3. *Elife* **7** (2018). https://doi.org:10.7554/eLife.42166

9 Han, Y. *et al.* High-yield monolayer graphene grids for near-atomic resolution cryoelectron microscopy. *Proceedings of the National Academy of Sciences* **117**, 1009-1014 (2020). https://doi.org:10.1073/pnas.1919114117

10 Punjani, A., Rubinstein, J. L., Fleet, D. J. & Brubaker, M. A. cryoSPARC: algorithms for rapid unsupervised cryo-EM structure determination. *Nature Methods* **14**, 290-296 (2017). https://doi.org:10.1038/nmeth.4169

11 Bepler, T. *et al.* Positive-unlabeled convolutional neural networks for particle picking in cryo-electron micrographs. *Nature Methods* **16**, 1153-1160 (2019). https://doi.org:10.1038/s41592-019-0575-8

12 Wagner, T. *et al.* SPHIRE-crYOLO is a fast and accurate fully automated particle picker for cryo-EM. *Commun Biol* **2**, 218 (2019). https://doi.org:10.1038/s42003-019-0437-z

13 Punjani, A., Zhang, H. & Fleet, D. J. Non-uniform refinement: adaptive regularization improves single-particle cryo-EM reconstruction. *Nat Methods* **17**, 1214-1221 (2020). https://doi.org:10.1038/s41592-020-00990-8

14 Sanchez-Garcia, R. *et al.* DeepEMhancer: a deep learning solution for cryo-EM volume post-processing. *Communications Biology* **4**, 874 (2021). https://doi.org:10.1038/s42003-021-02399-1

15 Punjani, A. & Fleet, D. J. 3DFlex: determining structure and motion of flexible proteins from cryo-EM. *Nature Methods* **20**, 860-870 (2023). https://doi.org:10.1038/s41592-023-01853-8

16 Jumper, J. *et al.* Highly accurate protein structure prediction with AlphaFold. *Nature* **596**, 583-589 (2021). https://doi.org:10.1038/s41586-021-03819-2

17 Mirdita, M. *et al.* ColabFold: making protein folding accessible to all. *Nature Methods* **19**, 679-682 (2022). https://doi.org:10.1038/s41592-022-01488-1

18 Emsley, P., Lohkamp, B., Scott, W. G. & Cowtan, K. Features and development of Coot. *Acta Crystallographica Section D Biological Crystallography* **66**, 486-501 (2010). https://doi.org:10.1107/s0907444910007493

19 Liebschner, D. *et al.* Macromolecular structure determination using X-rays, neutrons and electrons: recent developments in Phenix. *Acta Crystallographica Section D* **75**, 861-877 (2019). https://doi.org:doi:10.1107/S2059798319011471

20 Croll, T. I. ISOLDE: a physically realistic environment for model building into low-resolution electron-density maps. *Acta Crystallogr D Struct Biol* **74**, 519-530 (2018). https://doi.org:10.1107/s2059798318002425

21 Pettersen, E. F. *et al.* UCSF ChimeraX: Structure visualization for researchers, educators, and developers. *Protein Sci* **30**, 70-82 (2021). https://doi.org:10.1002/pro.3943

22 Rohou, A. & Grigorieff, N. CTFFIND4: Fast and accurate defocus estimation from electron micrographs. *Journal of Structural Biology* **192**, 216-221 (2015). https://doi.org:https://doi.org/10.1016/j.jsb.2015.08.008

23 Grant, T., Rohou, A. & Grigorieff, N. cisTEM, user-friendly software for single-particle image processing. *eLife* **7**, e35383 (2018). https://doi.org:10.7554/eLife.35383

24 Ben Chorin, A. *et al.* ConSurf-DB: An accessible repository for the evolutionary conservation patterns of the majority of PDB proteins. *Protein Sci* **29**, 258-267 (2020). https://doi.org:10.1002/pro.3779

25 Madeira, F. *et al.* Search and sequence analysis tools services from EMBL-EBI in 2022. *Nucleic acids research* **50**, W276-W279 (2022). https://doi.org:10.1093/nar/gkac240
